# Unravelling the Influence of the Local Structure on the Ultralow Thermal Conductivity of the Bismuthinite–Aikinite Series, Cu_1–*x*_□_*x*_Pb_1–*x*_Bi_1+*x*_S_3_

**DOI:** 10.1021/jacs.5c12526

**Published:** 2025-10-01

**Authors:** Paz Vaqueiro, Anna Herlihy, Mahmoud Elgaml, Shriparna Mukherjee, David A. Keen, David J. Voneshen, Anthony V. Powell

**Affiliations:** † Department of Chemistry, 6816 University of Reading, Whiteknights, Reading RG6 6DX, U.K.; ‡ 120796 Diamond Light Source, Harwell Science and Innovation Campus, Didcot OX11 0DE, U.K.; § ISIS Pulsed Neutron and Muon Source, Rutherford Appleton Laboratory, Harwell Campus, Didcot OX11 0QX, U.K.; ∥ Department of Physics, Royal Holloway University of London, Egham, Surrey TW20 0EX, U.K.

## Abstract

Understanding the relationship between crystal structure, bonding and thermal transport is critical for the discovery of materials with ultralow thermal conductivities. Materials in the bismuthinite–aikinite series, Cu_1–*x*_□_*x*_Pb_1–*x*_Bi_1+*x*_S_3_ (0 ≤ *x* ≤ 1), in which a Bi^3+^ cation and a vacancy (□) are progressively substituted by a Pb^2+^ and a Cu^+^ cation, exhibit ultralow thermal conductivities (∼0.5 W m^−1^K^–1^ for *x* < 1). Here, we investigate the effect of decreasing the Pb^2+^ and Cu^+^ content on the crystal structure and properties of Cu_1–*x*_□_*x*_Pb_1–*x*_Bi_1+*x*_S_3_ (*x* = 0, 0.33, 0.6 and 0.83). These materials exhibit two-channel thermal transport, with non-propagating phonons being the dominant contribution. Neutron diffraction data reveal that intermediate compositions crystallize in the krupkaite structure (*x* = 0.5, *P*2_1_ *ma*), instead of the end-member aikinite structure (*x* = 0, *Pnma*). Pair distribution function (PDF) analysis reveals that the disordering of vacancies and cations deviates significantly from that expected for a statistical distribution and that, at a local level, copper-rich and copper-poor regions occur. Reducing the Pb^2+^ and Cu^+^ content results in lattice softening, which may be attributed to the increased concentration of vacancies in copper-poor regions. Moreover, the persistence of short Pb^2+^–Cu^+^ distances in the copper-rich regions is likely to facilitate the cooperative interaction between lone pairs and rattling Cu^+^ cations that leads to phonon scattering. These findings provide crucial insights into the effect of the local structure on the phonon transport and highlight the potential of local-structure design to achieve high thermoelectric performance in crystalline solids.

## Graphical abstract

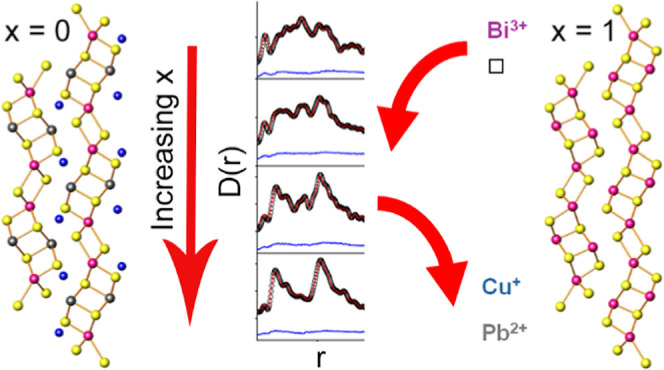

## Introduction

Thermoelectric energy recovery, which involves converting waste heat into useful electrical energy, is a promising technology that could make a significant contribution to the net-zero goal. The efficiency of thermoelectric energy recovery is related to a dimensionless figure of merit, *ZT* = (*S* ^2^σ*T*)/κ (where *S*, σ, *T* and κ are the Seebeck coefficient, electrical conductivity, absolute temperature and thermal conductivity, respectively) of the p- and n-type semiconducting materials found in a thermoelectric device.^1^ Since κ = κ_L_ + κ_e_ and the electrical conductivity, σ, is related to the electronic component of the thermal conductivity, κ_e_, by the Wiedemann–Franz law, many of the strategies to increase *ZT* focus on reducing the lattice thermal conductivity, κ_L_.^2^ Approaches related to the nature of the chemical bonding^3^ that can lead to significant reductions in κ_L_, include liquid-like ionic mobility,^4^ rattling,^5^ bonding heterogeneity^6^ and anharmonicity induced by lone pairs.^3^

As many of the high-performance thermoelectric materials contain scarce elements such as tellurium, the search for alternative materials, containing environmentally-friendly and Earth-abundant elements, is extremely active at present.^7^ In this context, sulfides are attractive as potential thermoelectric materials due to the large terrestrial abundance and availability of sulfur.^8^ While there are already several families of p-type sulfides with figures of merit that approach or exceed unity, such as Cu_2–*x*_S^9^ or tetrahedrites,^10^ the thermoelectric performance of n-type sulfides is significantly lower.^11^ Minerals in the bismuthinite–aikinite series, Cu_1–*x*_□_*x*_Pb_1–*x*_Bi_1+*x*_S_3_ (0 ≤ *x* ≤ 1), in which pairs of a Bi^3+^ cation and a vacancy (□) are substituted by pairs of Pb^2+^ and Cu^+^ cations,^12^ are attracting attention as potential n-type thermoelectric sulfides, owing to their ultralow thermal conductivities.^12–16^ All materials in this series are structurally related,^17^ as illustrated in Figure 1. The bismuthinite end member, Bi_2_S_3_ (*x* = 1), contains Bi_4_S_6_ ribbons in a herringbone pattern.^18^ In aikinite (*x* = 0), half of the Bi^3+^ cations are replaced with Pb^2+^ in an ordered fashion, with Cu^+^ cations filling tetrahedral interstitial sites between Bi_2_Pb_2_S_6_ ribbons.^19^ The composition *x* = 0.5, which corresponds to the mineral krupkaite, contains Bi_3_PbS_6_ ribbons, with half of the available tetrahedral sites occupied by Cu^+^ cations in an ordered fashion.^20^ Minerals with intermediate compositions exhibit ordered superstructures, containing varying amounts of bismuthinite, aikinite and krupkaite ribbons (Figure 1). They crystallize in the space groups *Pnma* or *P*2_1_ *ma* with *a*≈*a*_a_, *b*≈*b*_a_ and *c*≈*n* × *c*_a_, where *a*_a_ *, b*_a_ and *c*_a_ refer to the lattice parameters of aikinite, and *n* is a positive integer. By contrast, synthetic samples with intermediate compositions appear to adopt a disordered aikinite structure, in which copper and vacancies are randomly distributed over the available tetrahedral holes.^12,16,21^ Since structural studies on synthetic samples have been primarily performed using X-ray diffraction, which provides no contrast between the isoelectronic Pb^2+^ and Bi^3+^ cations, little is known about the degree of Pb^2+^/Bi^3+^ disorder.

**Figure 1:**
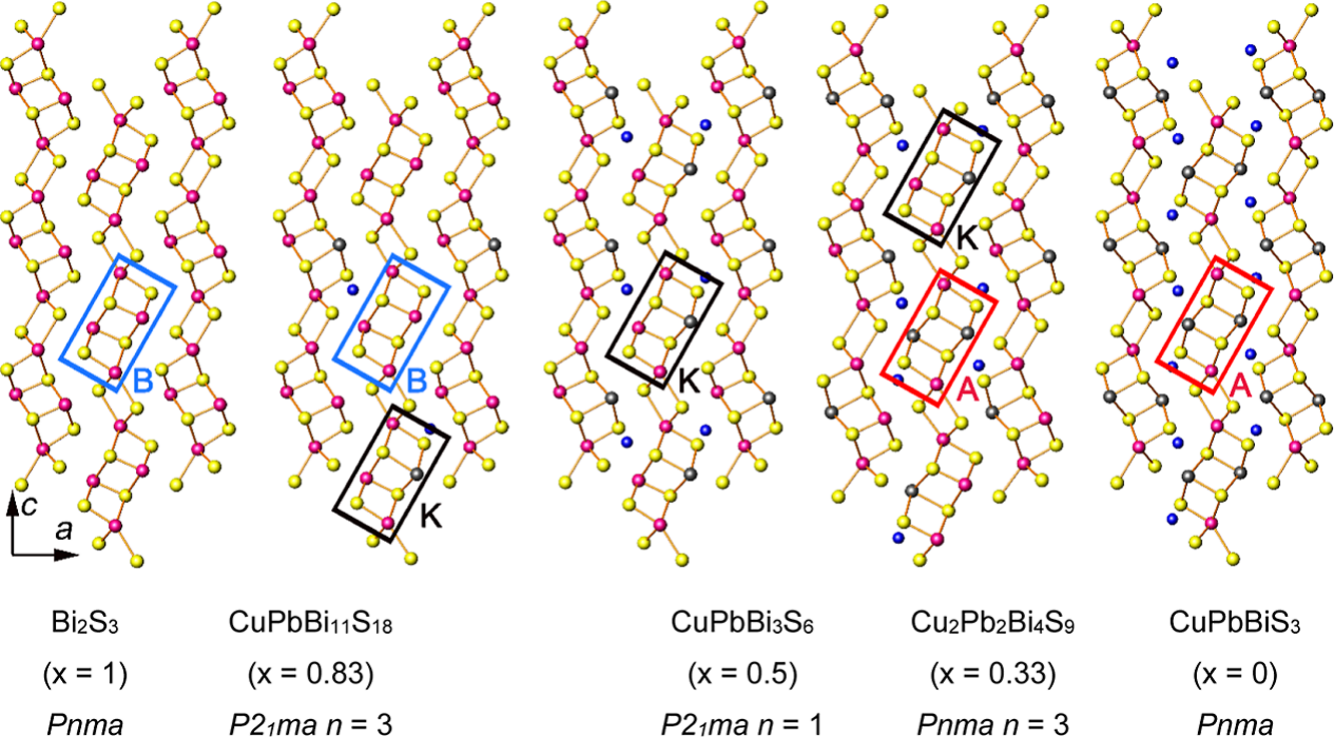
Comparison between the crystal structures of bismuthinite (*x* = 1), pekoite (*x* = 0.83), krupkaite (*x* = 0.5), hammarite (*x* = 0.33) and aikinite (*x* = 0), with selected aikinite (A), bismuthinite (B) and krupkaite (K) ribbons highlighted by red, blue and black rectangles, respectively. View along [010]. Key: copper, blue spheres; bismuth, pink spheres; lead, dark gray spheres; sulfur, yellow spheres.

Most of the studies of the thermoelectric properties of Cu_1–*x*_□_*x*_Pb_1–*x*_Bi_1+*x*_S_3_ have been performed on the bismuthinite end member (*x* = 1). Doping of Bi_2_S_3_ with halides, through the incorporation of dopants such as BiCl_3,_ SbCl_3_, InCl_3_ and HfCl_4_, leads to significant increases in the electrical conductivity and the thermoelectric figure of merit.^22–25^ Dopants can also increase phonon scattering through increased grain boundaries and the formation of nanoprecipitates.^25^ Doping of Bi_2_S_3_ with copper has resulted in *ZT* = 0.62 at 723 K for Cu_0.01_Bi_2_S_3_,^26^ while silver and chlorine co-doping leads to *ZT* ≈ 0.9 at 676 K.^27^ Reports of the thermoelectric properties of other members of the bismuthinite–aikinite series appear to be limited to CuPbBi_5_S_9_ (*x* = 2/3),^12,14^ Cu_0.14_Pb_0.14_Bi_1.86_S_3_ (*x* = 0.14)^16^ and CuPbBiS_3_ (*x* = 0).^13^ For instance, it has been shown that Cl doping in CuPbBi_5_S_9_ (*x* = 2/3) leads to *ZT* = 0.43 at 700 K,^12^ while undoped Cu_0.14_Pb_0.14_Bi_1.86_S_3_ (*x* = 0.14) achieves *ZT* = 0.21 at only 475 K.^16^

Here, we sought to investigate the effect of varying the copper and lead content on the structure and the thermoelectric properties of Cu_1–*x*_□_*x*_Pb_1–*x*_Bi_1+*x*_S_3_ (0 ≤ *x* ≤ 1). We have recently shown that the ultralow thermal conductivity of crystalline aikinite, CuPbSbS_3_ (*x* = 0), which is close to the calculated minimum for amorphous materials, is a consequence of a cooperative interaction between the rotating Pb^2+^ lone pair and the Cu^+^ cations.^13^ The results presented here show that decreasing the copper and lead content results in lattice softening, arising from the formation, at a local level, of copper-poor regions containing large numbers of vacancies. Moreover, in the copper-rich regions, short Pb^2+^–Cu^+^ distances persist, and can facilitate the cooperative interaction between lone pairs and rattling Cu^+^ cations that leads to phonon scattering.

## Experimental Details

Samples with composition Cu_1–*x*_□_*x*_Pb_1–*x*_Bi_1+*x*_S_3_, where *x* = 0, 0.33, 0.6 and 0.83, corresponding to the stoichiometries of the minerals aikinite,^19^ hammarite,^28^ paarite^29^ and pekoite^21^ respectively, were prepared by ball milling. Stoichiometric amounts of Cu (Sigma-Aldrich, 425 μm powder, 99.5%), Pb (Goodfellow, rods 3.2 mm dia., 99.95%), Bi (Alfa Aesar, needles, 99.99%) and S (Sigma-Aldrich, flakes, 99.99%) were loaded into a 45 mL stainless-steel jar inside a glovebox under an Ar atmosphere. Prior to loading into the jar, the Pb rods were cut into small pieces and the Bi needles were ground into a powder using a pestle and mortar under an Ar atmosphere. Eighteen stainless-steel balls, each with a diameter of 10 mm, were added to the stainless-steel jar, prior to sealing it, under an Ar atmosphere. This resulted in a powder-to-ball weight ratio of 1:12. Milling was carried out using a Fritsch Pulverisette 6 Planetary Ball Mill at 500 rpm, for 60 h. Milling was intermittent: it was stopped for 10 min after every 10 min of milling to avoid overheating the sample. Following milling, the resulting powders were sealed into evacuated (<10^–4^ mbar) fused-silica ampules. The sealed ampules were heated to 573 K (at a rate of 1 K min^–1^), held for 48 h at this temperature and subsequently cooled to room temperature (at a rate of 1 K min^–1^). The annealed powders were hand ground in air and consolidated into densified pellets of ∼13 mm diameter, by hot pressing under N_2_ at 523 K under 80 MPa for 1 h. After releasing the pressure, the hot press was cooled for approximately 1 h to room temperature, with the sample still under a N_2_ atmosphere. The density of each hot-pressed pellet was determined using the Archimedes’ method, using an AE Adam PW 184 balance. The prepared pellets have densities ranging between 6.3 and 6.9 g cm^–3^, which are >90% of the corresponding crystallographic densities.

Samples were initially characterized by powder X-ray diffraction. Diffraction patterns were collected using a Bruker D8 Advance Powder X-ray diffractometer, operating with Ge-monochromated Cu K_α1_ (λ = 1.54046 Å) radiation. Rietveld refinements, to determine the lattice parameters (Supporting Information, Table S1), were performed using the GSAS software.^30^ The electrical conductivity and Seebeck coefficient were measured simultaneously using a Linseis LSR 3, under a helium atmosphere. Graphite-coated circular pellets with a diameter of 12.7 mm and thickness ∼1.5–2 mm were used for the thermal diffusivity (*D*) measurements using a Netzsch LFA 447 NanoFlash system. The total thermal conductivity (κ) was calculated taking into account that κ = *DdC*_*p*_ where *d* is the density of the material and *C*_*p*_ is the specific heat capacity. The Dulong-Petit limit for *C*_*p*_ was used for the calculation of κ. The Lorenz number *L* was determined using the relation *L* = 1.5 + exp­(−*|S|*/116), where *S* is the temperature dependent Seebeck coefficient.^31^ The weighted mobility was calculated from the measured electrical resistivity and Seebeck coefficient values,^32^ as described in the Supporting Information. The electronic component of the thermal conductivity, κ_e_, was calculated using the Wiedemann–Franz relation, and the lattice component as the difference between the total thermal conductivity and the electronic component. The transverse (*v*_T_) and longitudinal (*v*_L_) sound velocities were measured using an Olympus 38DL Plus ultrasonic flaw detector with a transducer frequency of 5 MHz. The calculations of the elastic properties derived from the sound velocity measurements, as well as the calculations of the minimum lattice thermal conductivity are described in the Supporting Information. Differential scanning calorimetry (DSC) data were collected using a TA-Q2000 DSC instrument with a heating rate of 10 K min^–1^.

For total neutron scattering measurements, samples of Cu_1–*x*_□_*x*_Pb_1–*x*_Bi_1+*x*_S_3_ (*x* = 0.0, 0.33, 0.6, 0.83) were loaded into 8 mm cylindrical vanadium cans and measured using the General Materials Diffractometer (GEM) instrument^33^ at the ISIS Neutron and Muon Source for 900 μAh (∼6 h). Data^34^ were reduced using the Mantid software package^35^ and Rietveld refinements against the neutron diffraction data were performed using TOPAS version 7.^36^

The raw total scattering data were reduced using the GudrunN software^37^ (*Q*_max_ = 30 Å^–1^) to remove instrument and container backgrounds. Instrument and container backgrounds were measured separately and subtracted from the measured sample data. The normalized data were then Fourier transformed into PDFs for analysis and modeling.

Small-box PDF modeling was carried out using TOPAS Academic version 6 ^36^ and initial structures generated from Rietveld refinements of the diffraction patterns. Reverse Monte Carlo (RMC) refinements were performed using the RMCProfile software^38^ with the aim of obtaining information about cation clustering. The refinements used an 8 × 23 × 8 supercell of the unit cell determined by Rietveld analysis (producing near-cubic supercell dimensions). Atomistic configurations were generated by randomly replacing Cu atoms with vacancies (□), and Pb with Bi atoms according to their occupancies. Atom swapping between Cu and □, and between Pb and Bi atoms was allowed with a probability of 0.1. Minimum and maximum distance restraints were applied to the S–S, S–Cu/□ and S–Pb/Bi atom–atom distances to prevent unphysical bond distances (details provided in the Supporting Information) and simultaneous fitting to reciprocal (*F*(*Q*)) space data was carried out to constrain the model to the long-range crystal structure.

## Results and Discussion

The powder X-ray diffraction patterns of all the Cu_1–*x*_□_*x*_Pb_1–*x*_Bi_1+*x*_S_3_ samples can be indexed on the basis of the orthorhombic unit cell of aikinite (space group *Pnma*, *c* ≈ *c*_a_). There is no evidence of additional reflections at large *d*-spacings corresponding to the ordered supercells found in the equivalent mineral phases, where *c* ≈ 3 × *c*_a_ for *x* = 0.33 (*Pnma*),^28^ *c* ≈ 5 × *c*_a_ for *x* = 0.6 (*Pnma*)^29^ and *c* ≈ 3 × *c*_a_ for *x* = 0.83 (*P*2_1_ *ma*).^21^ Rietveld refinements using an aikinite model, in which copper and vacancies are disordered over the available tetrahedral sites, result in excellent agreement between the observed and calculated data (Supporting Information, Figure S1). With the exception of aikinite, which contains trace amounts of PbS (ca. 1% wt.), powder X-ray diffraction data indicate that all samples are single phases.

In the structural model for aikinite,^13^ the Pb^2+^ and Bi^3+^ cations are fully ordered over two distinct crystallographic sites, M(1) (0.833, 1/4, 0.113) and M(2) (0.0177, 1/4, 0.682), respectively. As it is not possible to distinguish between isoelectronic Pb^2+^ and Bi^3+^ by X-ray diffraction, we collected room-temperature neutron diffraction data, which provides contrast between these two elements (*b*_Pb_ = 9.4; *b*_Bi_ = 8.5 fm). Similarly to our findings from the powder X-ray diffraction data, for the composition *x* = 0.83, there is no evidence of additional reflections at large *d*-spacings, therefore Rietveld refinements for this composition were performed using a disordered aikinite structural model. A model in which the occupancy of the Pb^2+^ and Bi^3+^ cations is refined at the M(1) site, with the M(2) site occupied by Bi^3+^ only, led to good agreement between the observed and calculated diffraction patterns (Figure 2 and Supporting Information). The refined structural parameters determined by refinement of an aikinite structural model against neutron diffraction data are presented in Table 1.

**Figure 2:**
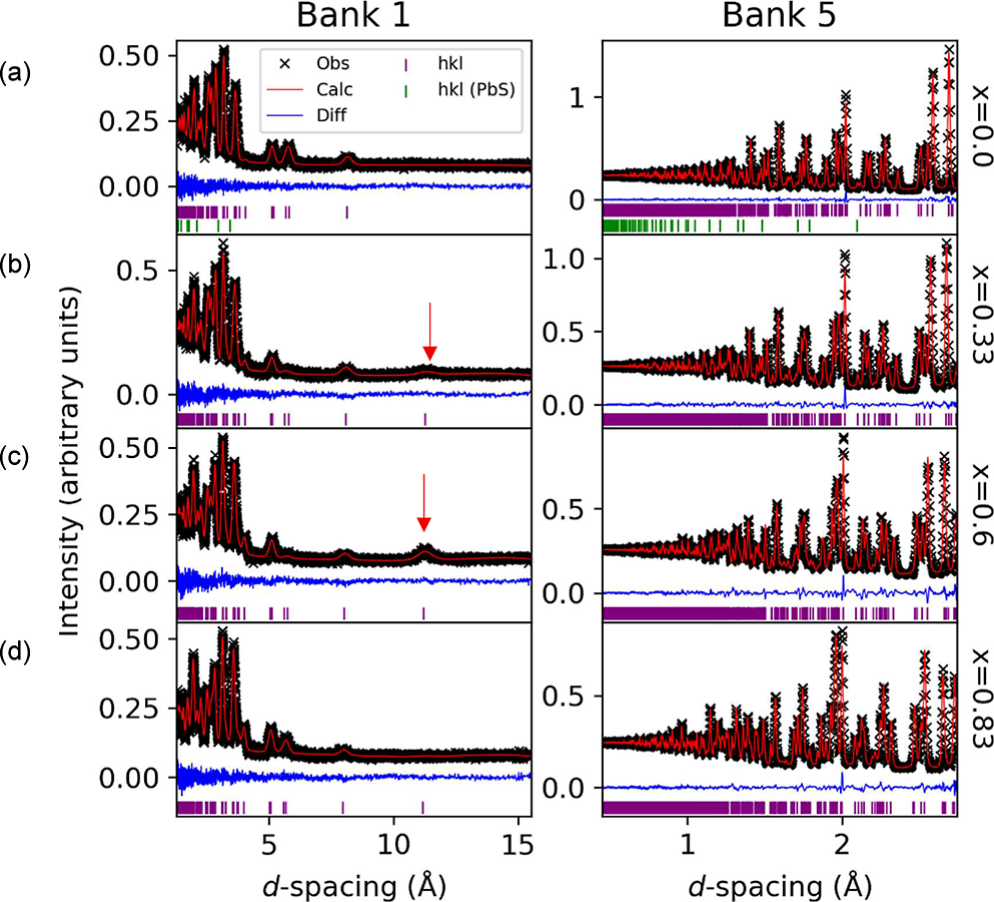
Rietveld refinements using neutron diffraction data (GEM detector banks 1 and 5, at 2θ = 9.4° and 91.4° respectively) collected on Cu_1–*x*_□_*x*_Pb_1–*x*_Bi_1+*x*_S_3_ at room temperature: (a) *x* = 0.0; (b) *x* = 0.33; (c) *x* = 0.6 and (d) *x* = 0.83. Reflection markers for the aikinite/krupkaite phase (pink) and for the PbS impurity found for *x* = 0.0 (green) are shown. Data for other banks can be found in the Supporting Information. The red arrow in (b,c) shows the position of the (001) reflection.

**Table 1:** Refined Parameters for Cu_1–*x*_□_*x*_Pb_1–*x*_Bi_1+*x*_S_3_ (*x* = 0.0, 0.83) (Space Group *Pnma*) Obtained from Rietveld Refinements Using Neutron Diffraction Data Collected at Room Temperature^a^

*x*		0	0.83
*a*/Å		11.6053(2)	11.3527(3)
*b*/Å		4.03998(7)	3.9931(1)
*c*/Å		11.3580(2)	11.1706(3)
M(1)	*x*	0.83321(6)	0.83901(7)
Pb/Bi	*z*	0.01131(6)	0.03083(6)
	SOF	1	0.17/0.83
	*B*/Å^2^	2.21(3)	2.03(3)
M(2)	*x*	0.01774(6)	0.01674(6)
Bi	*z*	0.68190(7)	0.6752(7)
	*B*/Å^2^	1.43(2)	1.45(3)
Cu	*x*	0.23462(7)	0.2264(5)
	*z*	0.20898(7)	0.2028(6)
	SOF	1	0.17
	*B*/Å^2^	1.97(3)	5.6(2)
S(1)	*x*	0.7134(2)	0.7150(2)
	*z*	0.6967(2)	0.6939(2)
	*B*/Å^2^	1.24(5)	1.03(6)
S(2)	*x*	0.0460(2)	0.0489(2)
	*z*	0.1382(2)	0.1286(2)
	*B*/Å^2^	1.16(5)	1.39(6)
S(3)	*x*	0.3783(2)	0.3768(2)
	*z*	0.0549(2)	0.0577(2)
	*B*/Å^2^	1.50(6)	0.99(6)
*R*_wp_/%		3.12	3.29

a All atoms occupy a 4c Wyckoff site, (*x*, 1/4, *z*). Unless otherwise specified, site occupancy factors (SOF) have a value of 1.0.

By contrast, examination of data collected for the compositions *x* = 0.33 and *x* = 0.6, revealed that, although the superlattice reflections that would be expected for *c* ≈ 3 × *c*_a_ or *c* ≈ 5 × *c*_a_ are absent, a reflection which can be indexed as (001), is present in the low-angle banks (Figure 2). This reflection is absent in the *Pnma* space group of aikinite (*x* = 0, *c* ≈ *c*_a_), but allowed in the *P*2_1_ *ma* space group of krupkaite (*x* = 0.5, *c* ≈ *c*_a_). Refinements were performed using structural models based on both crystal structures. In the crystal structure of krupkaite,^20^ there are two partially-occupied crystallographic sites for copper, and four distinct crystallographic sites in the M_4_S_6_ ribbons, of which one is occupied by lead and three by bismuth. In the Rietveld refinements using the krupkaite structural model, the copper occupancy was allowed to refine between the two available sites, with the constraint that the overall composition was maintained. Different models were tested for the distribution of lead between the four available sites in the M_4_S_6_ ribbons. The best agreement between observed and calculated patterns was obtained when the M(1) and M(2) sites were occupied only by bismuth. Therefore, the lead occupancy was refined at the M(3) and M(4) sites; this converged to structural models in which the M(3) site is fully occupied by bismuth for *x* = 0.6 and the M(4) site is fully occupied by lead for *x* = 0.33. For these compositions, refinements using a krupkaite model, presented in Table 2, resulted in significantly lower *R*_wp_ factors than those using an aikinite model. For instance, for *x* = 0.6, the final *R*_wp_ using the aikinite model is 4.11%, while the krupkaite model results in a *R*_wp_ value of 3.67%. Since these results indicate that some ordering of the Pb^2+^ and Cu^+^ cations occur for intermediate compositions, Rietveld refinements were carried out using a krupkaite model for *x* = 0.83 (Table 2). Despite the increase in the number of parameters, this resulted in only a slight reduction in the value of *R*_wp_.

**Table 2:** Refined Parameters for Cu_1–*x*_□_*x*_Pb_1–*x*_Bi_1+*x*_S_3_ (*x* = 0.33, 0.6, 0.83) (Space Group *P*2_1_ *ma*) Obtained from Rietveld Refinements Using Neutron Diffraction Data Collected at Room Temperature^a^

*x*		0.33	0.6	0.83
*a*/Å		11.5490(3)	11.4648(4)	11.3528(3)
*b*/Å		4.0299(1)	4.0095(1)	3.99316(9)
*c*/Å		11.2561(3)	11.2012(4)	11.1705(3)
M(1)	*x*	0.4825(2)	0.4848(2)	0.4807(2)
Bi 2a	*z*	0.9335(2)	0.9276(3)	0.9287(3)
	*B*/Å^2^	1.70(3)	1.87(4)	1.50(3)
M(2)	*x*	0.4460(2)	0.4503(2)	0.4479(2)
Bi 2b	*z*	0.5750(2)	0.5746(3)	0.5784(3)
	*B*/Å^2^	1.70(3)	1.87(4)	1.50(3)
M(3)	*x*	0.6215(2)	0.6205(2)	0.6207(3)
Pb/Bi 2b	*z*	0.2230(2)	0.2115(2)	0.2124(3)
	SOF	0.34/0.66	0/1.0	0/1.0
	*B*/Å^2^	2.77(4)	2.27(4)	1.83(3)
M(4)	*x*	0.2914(2)	0.2942(2)	0.2993(3)
Pb/Bi 2a	*z*	0.2576(2)	0.2612(2)	0.2740(2)
	SOF	1.0/0	0.8/0.2	0.34/0.66
	*B*/Å^2^	2.77(4)	2.27(4)	1.83(3)
Cu(1)	*x*	0.6965(4)	0.6895(4)	0.699(2)
2a	*z*	0.4645(4)	0.4634(5)	0.479(2)
	SOF	0.802(4)	0.613(4)	0.166(7)
	*B*/Å^2^	1.89(5)	3.1(1)	4.5(3)
Cu(2)	*x*	0.7332(6)	0.732(2)	0.746(1)
2b	*z*	–0.0499(6)	–0.068(2)	–0.070(2)
	SOF	0.538(4)	0.187(4)	0.174(7)
	*B*/Å^2^	1.89(5)	3.1(1)	4.5(3)
S(1)	*x*	0.5785(6)	0.5873(7)	0.5808(7)
2b	*z*	0.7954(7)	0.7941(7)	0.8015(7)
S(2)	*x*	0.4155(9)	0.4207(7)	0.4164(8)
2b	*z*	0.1164(9)	0.1109(7)	0.1159(7)
S(3)	*x*	0.2569(8)	0.2613(7)	0.2573(7)
2b	*z*	0.4502(7)	0.4491(7)	0.4501(7)
S(4)	*x*	0.3325(7)	0.3388(7)	0.3332(8)
2a	*z*	0.6863(6)	0.6851(6)	0.6867(6)
S(5)	*x*	0.512(1)	0.5164(8)	0.5160(8)
2a	*z*	0.3870(9)	0.3736(8)	0.3720(8)
S(6)	*x*	0.6808(7)	0.6836(6)	0.6854(6)
2a	*z*	0.0638(7)	0.0594(7)	0.0622(7)
	*B*/Å^2^	1.44(4)	1.20(5)	1.03(4)
*R*_wp_/%		3.28	3.67	3.21

a As indicated, atoms occupy 2a and 2b Wyckoff sites, (*x*, 0, *z*) and (*x*, 1/2, *z*), respectively. Unless otherwise specified, site occupancy factors (SOF) have a value of 1.0. The atomic displacement parameters, B, for all sulfur sites were constrained to the same value, listed as a parameter for S(6). Similarly, B for the pairs M(1) and M(2), M(3) and M(4), and Cu(1) and Cu(2) have been constrained to the same value.

The lattice parameters decrease with decreasing copper and lead content (Figure 3a), while ordering of the Pb^2+^ cations in the M_4_S_6_ ribbons persists up to at least *x* = 0.6 (Figure 3b), with preferential occupancy of the M(4) site (Table 2) by the Pb^2+^ cations. For *x* = 0.83, refinements carried out using the krupkaite model also indicate a preference for the M(4) site, as illustrated in Figure 3b. For all compositions, the atomic displacement parameters of the Pb^2+^ and Cu^+^ cations are larger than those of Bi^3+^ and S^2–^, as previously observed for the aikinite end member (*x* = 0).^13^ In aikinite, each Pb^2+^ cation is surrounded by three Cu^+^ cations at distances of approximately 3.3 Å, which are smaller than the sum of their van der Waals’ radii.^39^ Similarly, each Cu^+^ cation is surrounded by three Pb^2+^ cations. The presence of Cu^+^ cations near Pb^2+^ cations and vice versa might be justified by Pauling’s second and third rules.^40^ The second rule states that local electroneutrality must be preserved. When a trivalent Bi^3+^ cation in Bi_2_S_3_ (*x* = 1) is replaced by a divalent Pb^2+^ cation there is a reduction in positive charge, hence the anions surrounding the divalent cation will violate local electroneutrality. This can be restored via the insertion of a Cu^+^ cation in a neighboring interstitial site. Examination of the coordination environment of Pb^2+^ and Cu^+^ cations (Figure 3c), reveals that three CuS_4_ tetrahedra share edges with a PbS_7_ capped octahedron, with two additional CuS_4_ tetrahedra linked by corner sharing (omitted in Figure 3c). Since, according to Pauling’s third rule, sharing polyhedral edges or faces instead of corners reduces the stability due to increased electrostatic repulsion between cations, it is preferable for the capped-octahedral site to be occupied by a divalent Pb^2+^ cation, rather than a trivalent Bi^3+^ cation. The BiS_6_ octahedra in aikinite are edge linked to only two CuS_4_ tetrahedra (Figure 3d), and the Bi^3+^–Cu^+^ distances are longer (>3.54 Å).

**Figure 3:**
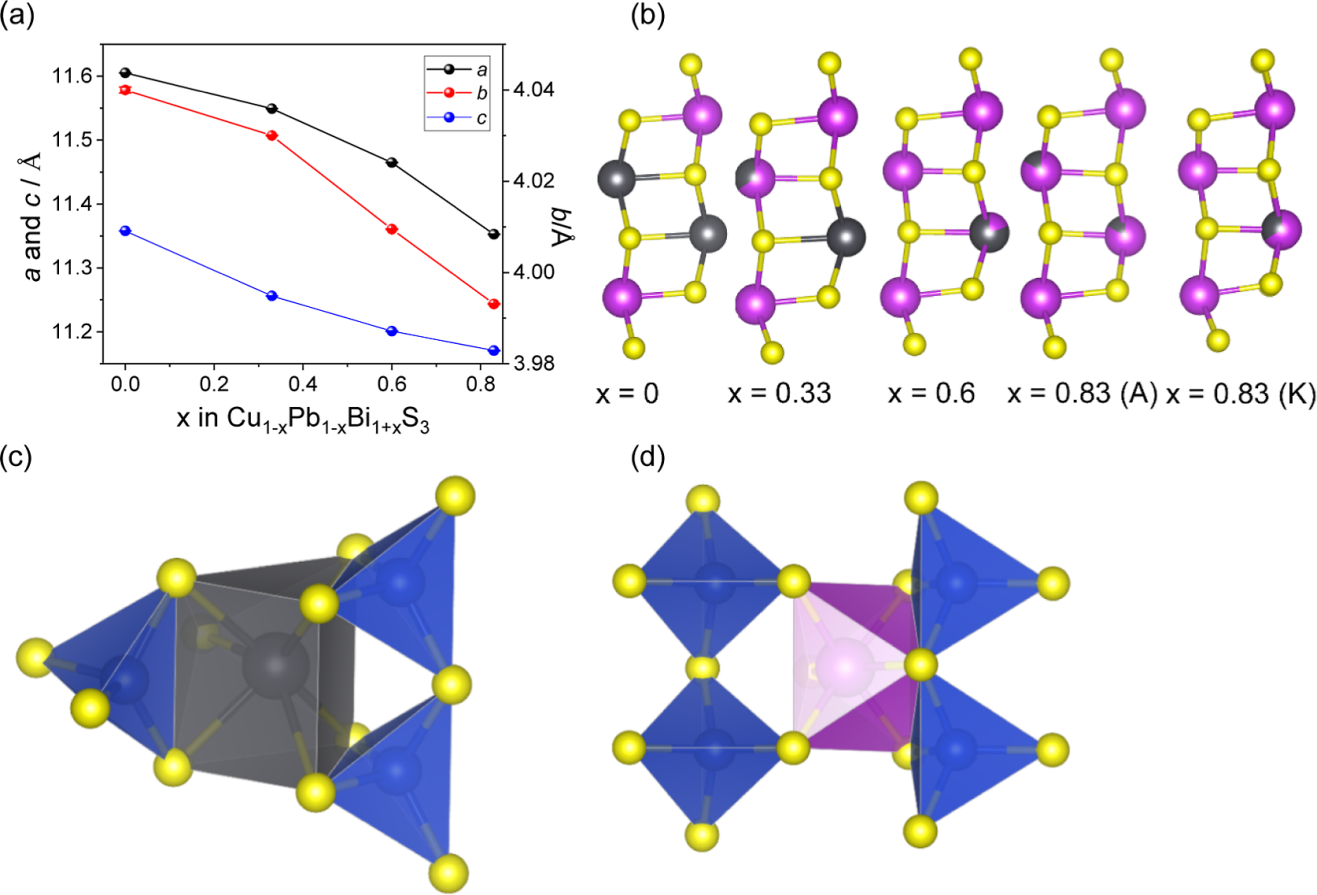
(a) Lattice parameters as a function of *x* for Cu_1–*x*_□_*x*_Pb_1–*x*_Bi_1+*x*_S_3_. Error bars are within the points. (b) The M_4_S_6_ ribbons, illustrating the ordering of Pb^2+^ cations for different values of *x*. For *x* = 0.83, the ribbons determined using an aikinite model (A) and a krupkaite model (K) are shown. (c) Linkage between CuS_4_ and PbS_7_ polyhedra in aikinite. Corner-sharing CuS_4_ tetrahedra have been omitted. (d) Linkage between CuS_4_ and BiS_6_ polyhedra in aikinite. Key as for Figure 1.

The Pb^2+^ and Cu^+^ cations are located in corrugated sheets parallel to the (100) planes (Figure 4a). With increasing *x*, vacancies and Bi^3+^ cations are introduced into these corrugated sheets. In the aikinite model, statistical mixing of Cu^+^ cations and vacancies around the M(1) site, which is mainly occupied by Pb^2+^ cations, can be described using a binomial distribution

$$P\left(n_{Cu}\right)=\frac{d!}{n_{Cu}!\left(d-n_{Cu}\right)!}\text{SO}F_{Cu}^{n_{Cu}}{\left(1-\text{SO}F_{Cu}\right)}^{\left(d-n_{Cu}\right)}$$

where *P*(*n*_Cu_) is the probability of having *n*_Cu_ nearest neighbors around the M(1) site, in a coordination shell containing *d* = 3 atoms, with SOF_Cu_ and (1 – SOF_Cu_) being the concentration of Cu^+^ cations and vacancies respectively. However, examination of these corrugated sheets (Figure 4b) reveals that, in the krupkaite model, the distribution of the Pb^2+^ and Cu^+^ cations differs from that in the aikinite model. In the krupkaite model, each M(4) site, which is preferentially occupied by Pb^2+^ cations, is surrounded by one cation/vacancy at the Cu(1) site and two cations/vacancies at the Cu(2) site, where the concentrations of Cu^+^ cations are given by SOF_1_ and SOF_2_ respectively. In this case, statistical analysis (Supporting Information, Table S2) shows that the probability of occurrence of a Pb^2+^ cation surrounded by vacancies, *P*(0), would be given by (1 – SOF_1_)­(1 – SOF_2_)^2^, while the probability of a Pb^2+^ cation being surrounded by 3 Cu^+^ cations, *P*(3), would be given by SOF_1_SOF_2_^2^. Comparison of the statistical probabilities between the aikinite and the krupkaite model as a function of the composition shows differences between both models when *x* < 0.83 (Figure 4c). In particular, a larger percentage of Pb^2+^ cations are surrounded by fewer Cu^+^ cations in the krupkaite model when compared to the aikinite model.

**Figure 4:**
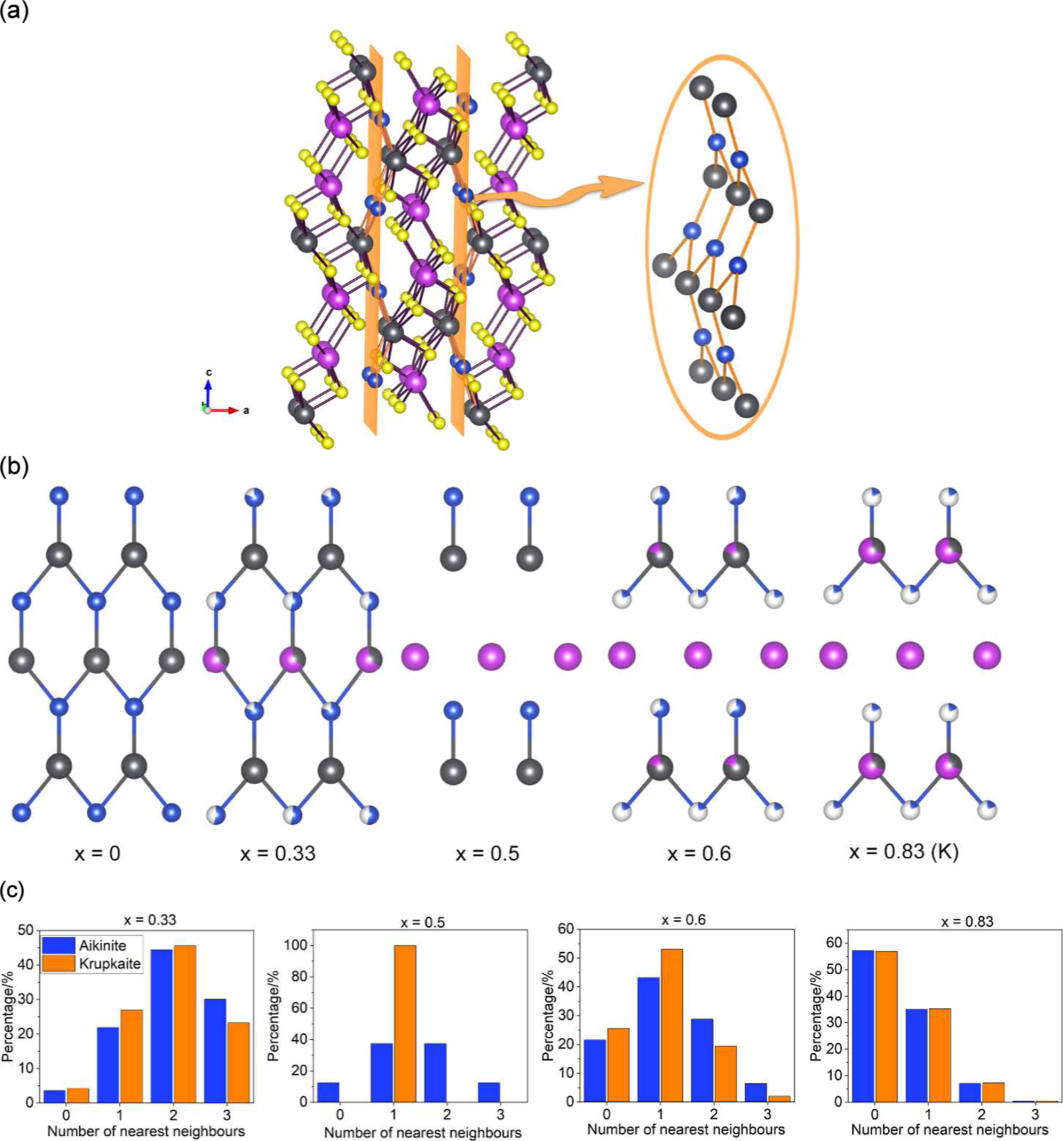
(a) Crystal structure of aikinite, highlighting the location of the Pb^2+^ and Cu^+^ corrugated sheets with orange planes. (b) The corrugated sheet formed by Pb^2+^ and Cu^+^ cations as a function of composition. Fully ordered krupkaite (*x* = 0.5)^41^ has been included for comparison purposes. For *x* = 0.83, the layer obtained using a krupkaite model is shown. View along [100]. Key as for Figure 1. (c) Calculated statistical distribution of the number of nearest Cu^+^ cation neighbors around the M(1) site in the aikinite model and around the M(4) site in the krupkaite model. M(1) and M(4) are the sites preferred by the Pb^2+^ cations in each model.

Since our previous work^13^ has shown that the interaction between the Pb^2+^ lone pair and the Cu^+^ cations has a marked effect on the phonon transport of the aikinite end member (*x* = 0), we investigated the local structure of the intermediate Cu_1–*x*_□_*x*_Pb_1–*x*_Bi_1+*x*_S_3_ compositions (0 < *x* < 1), using the neutron PDFs. Figure 5 shows representative small- and big-box refinements for the *x* = 0.83 composition (see Supporting Information, Figures S6–S8 for other compositions). Small-box modeling using the average aikinite structure yielded unsatisfactory refinements, with large values of *R*_wp_, ranging between 29.24 and 16.89%. The agreement is particularly poor for the shortest distances, which can be associated with Cu–S bonds. Small-box refinements for intermediate Cu_1–*x*_□_*x*_Pb_1–*x*_Bi_1+*x*_S_3_ compositions (0 < *x* < 1), using the krupkaite models determined from Rietveld refinements (Table 2) resulted in significantly lower *R*_wp_ values, ranging between 14.29 and 11.35%. However, unconstrained refinement of the atomic displacement parameters led to unrealistically large or small values (Supporting Information, Table S3). These unphysical atomic displacement parameters point toward the presence of disorder, which is not captured within the small-box model of a single krupkaite unit cell. Instead, big-box models, generated by expanding the krupkaite unit cell determined by Rietveld analysis to a supercell containing 35328 atoms, were fitted using the RMC method. In the initial supercell model, Cu^+^ and vacancies (□), and Pb^2+^ and Bi^3+^ cations were placed randomly in the disordered crystallographic sites: Cu(1) and Cu(2), and M(3) and M(4), respectively. In the initial stages of the fitting, only atom moves were allowed, with distance restraints applied (Supporting Information, Table S4). Later, atom swapping between Cu and □, and between Pb and Bi atoms was also allowed. For *x* = 0, the big-box RMC refinement was performed using only the aikinite structure and atom swapping was not allowed. The big-box models resulted in excellent agreement with the experimental data (Figures 5a and S8). For all intermediate compositions with 0 < *x* < 1, atom swapping resulted in lower **χ** ^2^ values (Supporting Information, Table S5) compared to models where swapping was not allowed. Partial occupancies for each crystallographic site containing Cu/□ or Pb/Bi, were calculated and were found to be comparable to those determined using Rietveld refinements.

**Figure 5:**
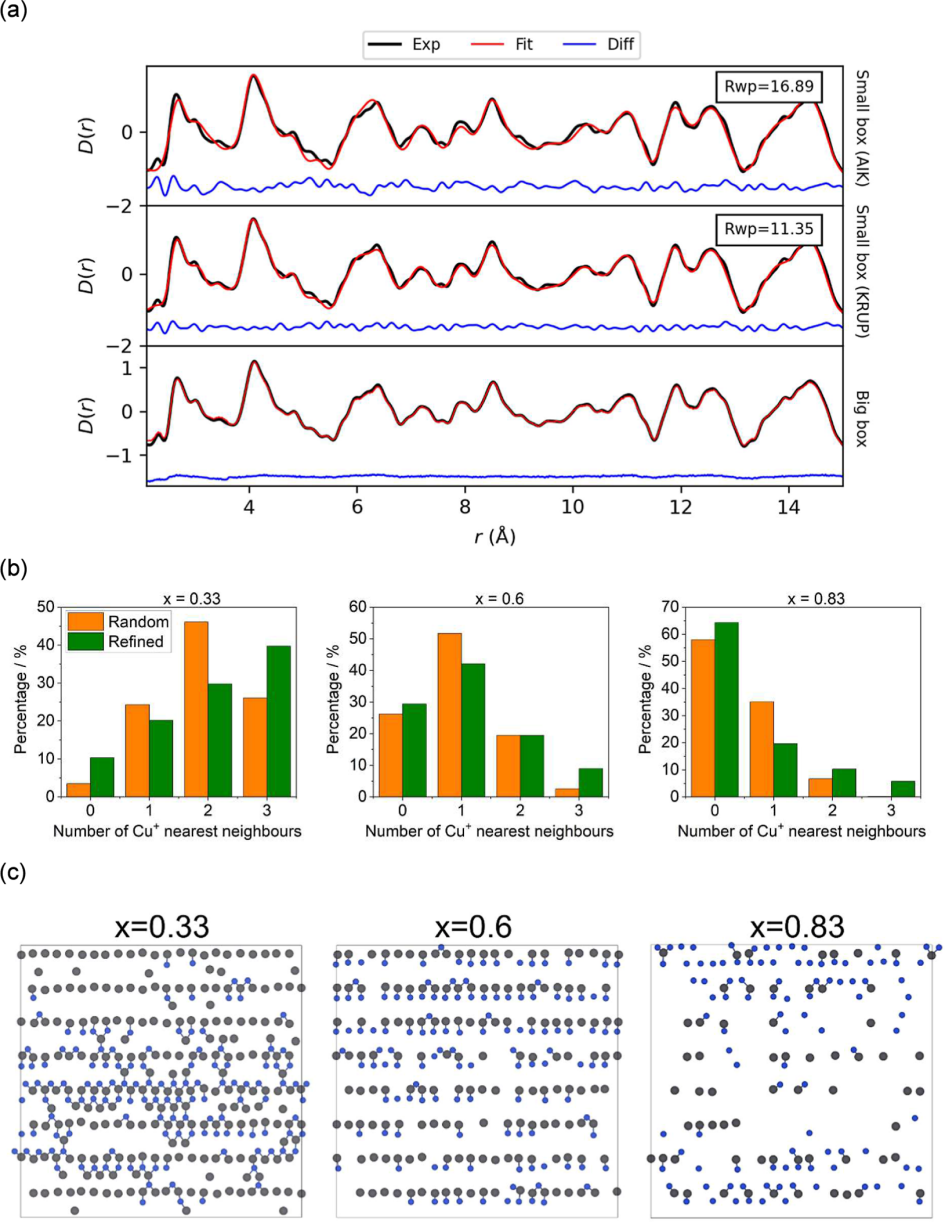
(a) Small-box and RMC refinements for Cu_1–*x*_□_*x*_Pb_1–*x*_Bi_1+*x*_S_3_ (*x* = 0.83) using neutron PDFs measured at room temperature. Top: small-box refinement using an aikinite model; middle: small-box refinement using a krupkaite model; bottom: RMC big-box refinement. (b) Number of Cu^+^ nearest neighbors around Pb^2+^ cations, determined from the initial (random) and refined big-box models of Cu_1–*x*_□_*x*_Pb_1–*x*_Bi_1+x_S_3_, using a krupkaite structural model. (c) View along [100] of selected sheets formed by Pb^2+^ (gray) and Cu^+^ (blue) cations as a function of composition, extracted from the refined big-box models. Bi^3+^ cations have been omitted for clarity.

Comparison and interrogation of the models allows for a more in-depth understanding of the local structure of the aikinite–bismuthinite series. The number of Cu^+^ nearest neighbors around each Pb^2+^ cation was compared to the statistical distribution of randomly placed atoms in the initial supercell model (Figure 5b). The distribution of randomly placed atoms in the initial supercell model, shown as orange bars in Figure 5b, is in good agreement with that calculated using a binomial distribution, shown as orange bars in Figure 4b. For all intermediate compositions with 0 < *x* < 1, atom swapping results in an increase in the percentages of Pb^2+^ cations with either no Cu^+^ neighbors or with three Cu^+^ neighbors and a corresponding decrease in Pb^2+^ cations with only one Cu^+^ neighbor. By contrast, examination of the number of Pb^2+^ neighbors around each Cu^+^ cation (Supporting Information, Figure S9) reveals a decrease in the percentage of Cu^+^ cations with no Pb^2+^ neighbors and an increase in Cu^+^ cations with at least one Pb^2+^ neighbor. Inspection of two-dimensional slices of the big boxes, parallel to the (100) planes (Figure 5c), unveils the presence of copper-poor and copper-rich areas. Locally, the corrugated sheets formed by the Pb^2+^ and Cu^+^ cations, resemble those found in bismuthinite in the copper-poor areas, and in aikinite and/or krupkaite in the copper-rich areas. The approximate dimensions of the copper-poor regions (Supporting Information, Table S10) were estimated by measuring the size of areas containing no copper on up to 16 slices, parallel to the (100) planes, for each composition and determining the average value. While for a given composition there is a broad distribution of sizes, the average copper-poor region size increases with increasing *x*, from an average value of 25 × 15 Å^2^ for *x* = 0.33 to 55 × 30 Å^2^ for *x* = 0.83. For all intermediate compositions, the copper-poor areas are larger than a single unit cell, which is approximately 4 × 11.2 Å^2^. Similarly, the size of the copper-rich regions was estimated by measuring the size of areas containing significant amounts of copper in up to 16 slices, parallel to the (100) planes (Table S10). The copper-rich areas increase in size with decreasing *x*.

The electrical transport properties of Cu_1–*x*_□_*x*_Pb_1–*x*_Bi_1+*x*_S_3_ as a function of temperature are presented in Figure 6. In all cases, the electrical conductivity increases with increasing temperature, while |*S*| decreases with increasing temperature, indicating that these materials are nondegenerate semiconductors. The low values of the electrical conductivity are consistent with previous reports for other materials in this series,^12,13^ with larger reported values often associated with the presence of secondary phases, such as bismuth metal.^14^ At a given temperature, the electrical conductivity increases with increasing *x*. There is considerable hysteresis in the electrical transport properties for samples with *x* > 0 (Supporting Information, Figure S10). Moreover, for *x* = 0.33 and 0.6, the electrical conductivity on initial heating shows anomalies between 450 and 500 K, which become weaker in subsequent measurements. It should be noted that the cooling rate during property measurements is faster than that applied during synthesis and consolidation. According to the tentative phase diagram for Cu_1–*x*_□_*x*_Pb_1–*x*_Bi_1+*x*_S_3_ proposed by Mumme and Watts, exsolution, accompanied by an order–disorder transition, is likely to occur around 500 K.^21^ This suggests that the anomalies in the electrical transport properties might have a structural origin, and is consistent with DSC data (Supporting Information, Figure S10) which shows a broad feature between 450 and 500 K.

**Figure 6:**
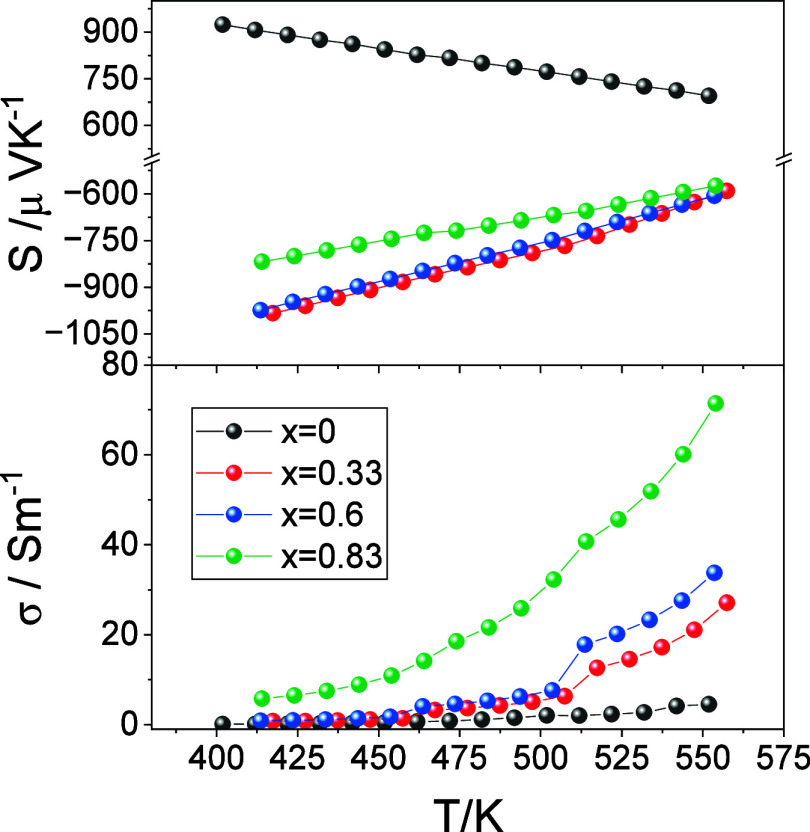
Temperature dependence of the electrical conductivity and Seebeck coefficient for Cu_1–*x*_□_*x*_Pb_1–*x*_Bi_1+*x*_S_3_ (*x* = 0.0, 0.33, 0.6, 0.83).

While samples with *x* > 0 exhibit a negative Seebeck coefficient, indicating that the dominant charge carriers are electrons, CuPbBiS_3_ (*x* = 0) is a p-type semiconductor (Figure 6). Electronic band structure calculations for CuPbBiS_3_ indicate that the main contributors to the top of the valence band are copper and sulfur states.^13^ In the crystal structure of CuPbBiS_3_ there is a continuous one-dimensional network of corner-sharing [CuS_4_]^7–^ tetrahedra which facilitates p-type electrical conduction. By contrast, other members of the bismuthinite–aikinite series, Cu_1–*x*_□_*x*_Pb_1–*x*_Bi_1+*x*_S_3_, with *x* > 0, contain vacancies in the one-dimensional [Cu_1–*x*_□_*x*_S_3_]^(5+*x*)–^ chains, instead of a continuous network. Therefore, in materials with *x* > 0, n-type electrical conduction involves the Bi–S network, as previously discussed by Maji et al., and arises due to the likely presence of sulfur vacancies.^12^ The weighted mobility, μ_w_, grows with increasing *x*. In particular, the weighted mobility of p-type CuPbBiS_3_ (*x* = 0), 1.24 cm^2^ V^–1^ s^–1^ at 410 K, is noticeably lower than the weighted mobilities of the n-type phases, which range between 18.79 and 20.67 cm^2^ V^–1^ s^–1^ at the same temperature. For Bi_2_S_3_ doped with 0.5% BiCl_3_, a slightly higher weighted mobility of 27.2 cm^2^ V^–1^ s^–1^ at 323 K has been reported.^27^

All measured materials in the Cu_1–*x*_□_*x*_Pb_1–*x*_Bi_1+*x*_S_3_ series exhibit an ultralow thermal conductivity (Figure 7a), of the order of 0.5 W m^–1^ K^–1^, which is dominated by the lattice component (Supporting Information, Figure S11). By contrast, Bi_2_S_3_ (*x* = 1) exhibits a much larger total thermal conductivity of ∼0.9 W m^–1^ K^–1^ at room temperature (Figure 7b),^24,25^ suggesting that the presence of Cu^+^ and Pb^2+^ cations has a significant effect on phonon transport. Comparison of the phonon density of states of Bi_2_S_3_ (*x* = 1)^42^ with that of CuPbBiS_3_ (*x* = 0)^12^ reveals that the gap around 100 cm^–1^ present in Bi_2_S_3_, is filled in CuPbBiS_3_. It has been previously shown that bond heterogeneity,^3^ which leads to markedly different interatomic force constants, can close gaps in the phonon density of states, and results in substantial increases in the number of phonon scattering processes, hence leading to significant reductions in lattice thermal conductivity.^6^ Analysis of the electron localization function (ELF) for CuPbBiS_3_ (*x* = 0) shows clear evidence of bond heterogeneity,^13^ also consistent with the larger values of the atomic displacement parameters found here for the Cu^+^ and Pb^2+^ cations for all compositions (Tables 1 and 2).

**Figure 7:**
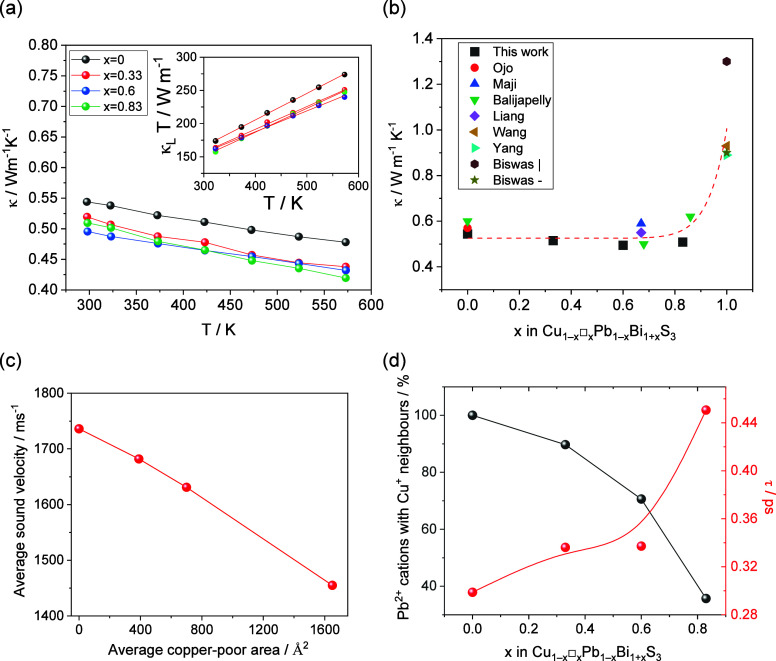
(a) Temperature dependence of the total thermal conductivity for Cu_1–*x*_□_*x*_Pb_1–*x*_Bi_1+*x*_S_3_ (*x* = 0.0, 0.33, 0.6, 0.83). Inset shows κ_L_ *T* vs *T*. Red lines are fits according to κ_L_ *T* = *A* + κ_0_ *T*. (b) The total thermal conductivity at room temperature as a function of composition. Data from the literature are shown.^12,14–16,22,24,25^ Measurements on highly oriented samples along the growth direction and perpendicular to it are labeled as | and – respectively. Red dashed line is a guide to the eye. (c) Relationship between the sound velocity and the area of the copper-poor regions. (d) Percentage of Pb^2+^ cations with one or more Cu^+^ neighbors and phonon lifetime, τ, as a function of composition. The phonon lifetime was estimated using the expression $\kappa_{L}=\frac{1}{3}C_{v}v_{a}^{2}\tau$ . Red and black lines are a guide to the eye.

The sound velocity (Table 3) decreases with decreasing copper and lead content. The elastic moduli, derived from the sound velocity measurements, follow the same trend and provide a measure of the bond strength. As previously observed for other thermoelectric materials,^43^ the elastic moduli of Cu_1–*x*_□_*x*_Pb_1–*x*_Bi_1+*x*_S_3_ (*x* < 1) decrease with increasing the average volume per atom (Supporting Information, Figure S12). Although in the Cu_1–*x*_□_*x*_Pb_1–*x*_Bi_1+*x*_S_3_ series the unit cell volume decreases with increasing *x*, the volume per atom increases from 22.2 Å^3^ for aikinite (*x* = 0) to 24.5 Å^3^ for *x* = 0.83 due to the introduction of vacancies in the Cu^+^ tetrahedral sites. This is likely to weaken the linkage between M_4_S_6_ ribbons along the [100] direction and hence result in lattice softening.

**Table 3:** Sound Velocities, Derived Elastic Properties and Calculated Minimum Lattice Thermal Conductivities (Cahill–Watson–Pohl κ_min,CWP_ and Diffusive κ_min,diff_) for Cu_1–*x*_□_*x*_Pb_1–*x*_Bi_1+*x*_S_3_

	*x* = 0	*x* = 0.33	*x* = 0.6	*x* = 0.83
transverse *v*_T_/m s^–1^	1560	1512	1466	1310
longitudinal *v*_L_/m s^–1^	2771	2688	2605	2283
average *v*_a_/m s^–1^	1736	1682	1631	1455
Poisson’s ratio	0.27	0.27	0.27	0.25
Young’s modulus/GPa	42.7	36.5	33.6	28.4
Shear modulus/GPa	16.8	14.4	13.2	11.36
Bulk modulus/GPa	30.9	26.4	24.3	18.9
Grüneisen parameter	1.59	1.59	1.59	1.52
Debye temp. θ_D_/K	183	176	169	149
κ_min,CWP_/W m^–1^ K^–1^	0.415	0.391	0.372	0.324
κ_min,diff_/W m^–1^ K^–1^	0.261	0.246	0.234	0.203

Considering that $\kappa_{L}=\frac{1}{3}C_{v}v_{a}l$ (where *C*_v_ is the heat capacity per unit volume and *l* is the phonon mean free path), we can estimate the phonon mean free path for these materials. This increases from approximately 5 Å for CuPbBiS_3_ (*x* = 0) to 6.5 Å for *x* = 0.83. The latter corresponds to approximately twice the interatomic spacing. The Grüneisen parameter, which changes very little across the series, is large (γ ≈ 1.6), indicating a high degree of anharmonicity, which is known to enhance phonon–phonon scattering. Indeed, the lattice thermal conductivity of these materials is close to the calculated minimum value (Table 3) using the Cahill–Watson–Pohl model (κ_min,CWP_), which is based on the phonon mean free path. However, calculations based on purely diffusive thermal transport (κ_min,diff_), which has been proposed as a better estimate for the minimum thermal conductivity,^44^ result in lower values of the minimum thermal conductivity, and suggest that further reductions in the lattice thermal conductivity of the Cu_1–*x*_□_*x*_Pb_1–*x*_Bi_1+*x*_S_3_ phases might be possible. In the aikinite end member (*x* = 0), κ_L_ values approach κ_min,CWP_ due to the combination of the lone pair rotation on the Pb^2+^ ions, anharmonicity and bond heterogeneity.^13^ While κ_L_ changes little across the series for *x* < 1, the calculated values of the minimum lattice thermal conductivity decrease with increasing *x*. This is consistent with the increase in phonon-mean free path that occurs with reducing the copper and lead content.

The lattice thermal conductivity of the Cu_1–*x*_□_*x*_Pb_1–*x*_Bi_1+*x*_S_3_ phases does not follow the usual inverse linear temperature dependence (κ_L_ ∝ *T* ^–1^), expected for Umklapp phonon scattering in crystalline materials.^45^ Instead, κ_L_ data exhibit temperature dependences (Supporting Information, Figure S11b) between *T* ^–0.21^ and *T* ^–0.31^. For crystalline materials with ultralow thermal conductivities, the deviation from a κ_L_ ∝ *T* ^–1^ dependence has been attributed to the coexistence of two mechanisms for phonon transport: propagating (κ_pg_) and diffusive (κ_diff_) channels,^46,47^ with κ_L_ = κ_pg_ + κ_diff_. The propagating contribution can be described using the Peierls–Boltzmann transport model (phonon gas) for crystalline solids, while the diffusive contribution, also called the coherent contribution, represents the Allen-Feldmann model (diffusons) for disordered systems.^48^ The physical origin of this term lies in the existence of phonons with mean free paths below the Ioffe–Regel limit due to anharmonic scattering, not in point defect scattering.^49^ In the unified theory of thermal transport,^50^ at high temperatures κ_pg_ ∝ *T* ^–1^, while κ_diff_ increases with rising temperature, and therefore the overall temperature dependence is weaker than that predicted by the Boltzmann transport equation. It has been proposed that, for the analysis of experimental data, the diffusive component of the lattice thermal conductivity could be approximated as κ_diff_ = κ_0_ exp (−*E*/*T*), where *E* is the energy of the dominant phonon excitation.^51^ At high temperatures, when *E* ≪ *T*, exp (−*E*/*T*) → 1, and κ_diff_ ≈ κ_0_. Thus, we can express the lattice thermal conductivity as κ_L_ = *A*/*T* + κ_0_, and therefore plots of κ_L_ *T* vs *T* should be linear, as shown in the inset of Figure 7a. The estimated values for *A* and κ_0_ derived from these plots are presented in Table S11. Comparison of the κ_0_ values so obtained with those of κ_L_ suggests that above room temperature, non-propagating phonons are the main contributors to the thermal transport, although the diffusive component, κ_0_, decreases with increasing *x*. While electron–phonon scattering, which also results in a temperature-independent scattering term,^52^ could potentially be a contributor, the charge carrier density in Cu_1–*x*_□_*x*_Pb_1–*x*_Bi_1+*x*_S_3_ is very low^12,13^ and therefore electron–phonon scattering is unlikely to have an impact. It has been shown in other materials that the propagating contribution (κ_pg_) originates mainly from the low-frequency (acoustic) phonons, while higher frequency phonons, and in particular anharmonic flat branches, contribute to the diffusive channel (κ_diff_), which arises from the coupling of vibrational modes.^50^ Two-channel thermal transport has been found in materials which exhibit ultralow values of κ_L_ and contain rattlers, such as Tl_3_VSe_4_,^46^ α-MgAgSb^47^ and AgTlI_2_.^53^ The phonon dispersion curves for the end member aikinite (*x* = 0), show clear evidence of low-energy rattling modes,^13^ arising from the vibrations of the Pb^2+^ and Cu^+^ cations and recent computational work demonstrates that the two-channel model is applicable to aikinite, with the acoustic modes (<5 meV) being the main contributors to κ_pg_, while densely packed optical modes at higher energies contribute to κ_diff_.^54^ Our experimental results show that κ_diff_ ≈ κ_0_ decreases with *x*. We can rationalize this observation by considering that the energetic proximity of the optical phonon modes is important in the diffusive channel, with increases in the energetic spacing resulting in a decrease in κ_diff_.^55^ Since the number of optical modes is given by 3*N* – 3 (where *N* is the number of atoms in the primitive unit cell), and in Cu_1–*x*_□_*x*_Pb_1–*x*_Bi_1+*x*_S_3_, the number of atoms per unit cell changes from 24 for *x* = 0 to 20 for *x* = 1, increasing *x* will reduce the number of optical modes. Thereby this will increase their spacing, hence leading to a reduction in κ_diff_. The propagating contribution, κ_pg,_ = *A*/*T*, rises slightly with increasing *x*. Since the phonon mean free path is approximately twice the interatomic spacing, the boundaries between the copper-rich and copper-poor regions, which are considerably larger than the phonon mean free path, are not expected to influence κ_pg_ significantly. Given the anharmonicity of these materials, κ_pg,_ is likely to be dominated by phonon–phonon scattering.

In aikinite (*x* = 0), we previously found that, in addition to bond heterogeneity, the interaction between Cu^+^ cations and the rotating Pb^2+^ lone pair is a key contributor to the phonon scattering processes that lower the lattice thermal conductivity.^13^ This interaction takes place in the corrugated sheets illustrated in Figure 4a,b, where each Pb^2+^ cation is surrounded by three Cu^+^ cations and vice versa. Our investigation of the local structure (Figure 5) for intermediate compositions in the Cu_1–*x*_□_*x*_Pb_1–*x*_Bi_1+*x*_S_3_ series demonstrates that, at a local level, the presence of Pb^2+^ cations surrounded by two or three Cu^+^ neighbors, persist up to at least *x* = 0.83. This can facilitate coupling of rotating lone pairs and the vibrational motion of the Cu^+^ cations. Moreover, sound velocity measurements (Table 3) show that considerable lattice softening occurs with increasing *x*. At a local level, there are copper-poor regions containing large numbers of vacancies, which can weaken local bonding stiffness and lower the frequency of the acoustic phonons. Indeed, the sound velocity decreases linearly with the average area of the copper-poor regions (Figure 7c), due to the lattice softening arising from the weaker bonding stiffness in these regions. Since κ_L_ ∝ *v*_a_^*n*^,^56^ where *v*_a_ is the average sound velocity and 1 ≤ *n* ≤ 3, a decrease in sound velocity should result in a reduction in κ_L_, if the phonon scattering remained unchanged. However, with increasing *x*, the number of Pb^2+^ cations that have one or more Cu^+^ neighbors is substantially reduced (Figure 7d). This will reduce the number of phonon scattering events arising from the Pb^2+^–Cu^+^ interaction, hence leading to an increase in the phonon lifetime, as shown in Figure 7d. As a consequence of these two opposing contributions, the lattice thermal conductivity changes very little across the series for *x* < 1.

The results presented here highlight the potential of local-structure design to achieve high thermoelectric performance in crystalline solids. It is increasingly becoming apparent that “hidden” local atomic motifs within an average crystal structure can exert a dramatic influence on the lattice thermal conductivity.^57^ If two or more types of atoms and/or vacancies are disordered at a crystallographic site, short-range ordering, which will not be evident when using classical crystallographic techniques, may occur. For instance, in the defective half-Heusler Nb_1–*x*_CoSb, which exhibits an abnormally low thermal conductivity, diffuse bands in electron diffraction data have been interpreted as short-range ordering of vacancies.^58^ The excellent thermoelectric performance of high-entropy rocksalt chalcogenides, which contain multiple elements in the cation site, was initially attributed to large strain from a severely distorted lattice, with the reduction in κ_L_ deviating markedly from that expected based on conventional mass- and strain-field fluctuations.^59^ More recent work on high-entropy thermoelectric chalcogenides suggests that “local chemical fluctuations”, consistent with deviations from a random distribution of cations, occur.^60^ Our work on the Cu_1–*x*_□_*x*_Pb_1–*x*_Bi_1+*x*_S_3_ series demonstrates that PDF can provide key structural information. In particular, PDF analysis shows that the reduction in sound velocity can be correlated to the formation of copper-poor regions (Figure 7c), and that structural features that give rise to strong anharmonicity and increased phonon scattering in aikinite (i.e., the interaction between Pb^2+^ and Cu^+^ cations) persist up to high values of *x*, whereas if the cation distribution was random, this interaction should vanish. The observed thermal history dependence (Figure S10) of the electrical transport properties of Cu_1–*x*_□_*x*_Pb_1–*x*_Bi_1+*x*_S_3_ suggests that the cation distribution may change depending on the synthesis temperature and cooling rate. This could be exploited for the manipulation of the local structure in thermoelectric materials undergoing order–disorder phase transitions, in order to enhance the thermoelectric performance.

## Conclusions

The average crystal structure of intermediate compositions in the Cu_1–*x*_□_*x*_Pb_1–*x*_Bi_1+*x*_S_3_ series can be described using a krupkaite model (space group *P*2_1_ *ma*), with *a≈a*_a_, *b≈b*_a_ and *c≈c*_a_. However, PDF analysis shows that the disorder of vacancies, Cu^+^, Pb^2+^ and Bi^3+^ cations deviates significantly from that expected for a statistical distribution and that, at a local level, copper-rich and copper-poor regions occur for all intermediate compositions (*x* = 0.33, 0.6 and 0.83).

The transport properties reported here for the Cu_1–*x*_□_*x*_Pb_1–*x*_Bi_1+*x*_S_3_ series have been measured on nominally undoped compositions. Since the thermoelectric quality factor is proportional to μ_w_/κ_L_,^32^ and μ_w_ increases with *x* while κ_L_ changes little for *x* < 1, we would expect that the highest values of *ZT* in the Cu_1–x_□_*x*_Pb_1–x_Bi_1+x_S_3_ series would be found for samples with low copper and lead contents. Doping will be required in order to increase the charge carrier concentration, which in this family of materials is unusually low (10^12^ to 10^14^ cm^–3^) for nominally undoped compositions.^12,13^

Sound velocity measurements show that reducing the Cu^+^ and Pb^2+^ content results in lattice softening, which can be ascribed to the increased concentration of vacancies in the copper-poor regions. Moreover, the presence of copper-rich regions means that the presence of Pb^2+^ cations surrounded by two or three Cu^+^ neighbors persists up to high values of *x*. This is likely to facilitate the cooperative interaction between lone pairs and rattling Cu^+^ cations, first identified in aikinite,^13^ which leads to phonon scattering. These findings highlight the pivotal role of the local structure in reducing the lattice thermal conductivity of the Cu_1–*x*_□_*x*_Pb_1–*x*_Bi_1+*x*_S_3_ series.

## Supplementary Material



## Notes

Details of methodology for weighted mobility, sound velocity and minimum thermal conductivity, Rietveld refinements using X-ray and neutron diffraction data, small-box and big-box refinements, electrical and thermal transport data (PDF)
