# Supplementary material for: Unravelling the Influence of the Local Structure on the Ultralow Thermal Conductivity of the Bismuthinite–Aikinite Series, Cu1–x □ x Pb1–x Bi1+x S3
Source: J Am Chem Soc. 2025 Oct 1;147(41):37598–610. doi: 10.1021/jacs.5c12526 (PMC12532196; doi:10.1021/jacs.5c12526)
Supplement: Supplementary file 1 [file ja5c12526_si_001.pdf]

# Supporting Information for

# Unravelling the influence of the local structure on the ultralow thermal conductivity of the bismuthinite-aikinite series, $Cu_{1-x}\square_xPb_{1-x}Bi_{1+x}S_3$

Paz Vaqueiro,[a]* Anna Herlihy,[b] Mahmoud Elgaml,[a] Shriparna Mukherjee,[a] David A. Keen,[c] David J. Voneshen,[c,d] Anthony V. Powell[a]

[a] *Department of Chemistry, University of Reading, Whiteknights, Reading RG6 6DX, UK*

[b] *Diamond Light Source, Harwell Science and Innovation Campus, Didcot OX11 0DE, UK*

[c] *ISIS Pulsed Neutron and Muon Source, Rutherford Appleton Laboratory, Harwell Campus, Didcot OX11 0QX, UK*

[d] *Department of Physics, Royal Holloway University of London, Egham, Surrey, TW20 0EX, UK*

* Corresponding author: E-mail address: p.vaqueiro@reading.ac.uk

**Weighted mobility:** The following expression[1] was used to calculate the weighted mobility, $\mu_w$:

$$\mu_w = 331\,\frac{cm^2}{Vs}\left(\frac{m\Omega\,cm}{\rho}\right)\left(\frac{T}{300\,K}\right)^{-3/2}\left[\frac{exp\left[\frac{|S|}{k_B/e}-2\right]}{1+exp\left[-5\left(\frac{|S|}{k_B/e}-1\right)\right]}+\frac{\frac{3}{\pi^2}\frac{|S|}{k_B/e}}{1+exp\left[5\left(\frac{|S|}{k_B/e}-1\right)\right]}\right]$$

with the electrical resistivity, $\rho$, given in m$\Omega$ cm, the Seebeck coefficient, S, in $\mu$V K$^{-1}$ and $k_B/e$ = 86.4 $\mu$V K$^{-1}$.

**Sound velocity measurements**: The following expressions were used to extract elastic moduli, the Debye temperature and the Grüneisen parameter from the sound velocity measurements.

Average velocity

$$v_a = \left(\frac{1}{3}\left[\frac{1}{v_l^3}+\frac{2}{v_t^3}\right]\right)^{-1/3}$$

Poisson ratio

$$v_p = \frac{1-2(v_t/v_l)^2}{2-2(v_t/v_l)^2}$$

Grüneisen parameter

$$\gamma = \frac{3}{2}\left(\frac{1+v_p}{2-3v_p}\right)$$

Young's modulus

$$E = \frac{\rho v_l^2(1+v_p)(1-2v_p)}{(1-v_p)}$$

Shear modulus:

$$G = \frac{E}{2(1+v_P)}$$

Bulk modulus:

$$B = \frac{E}{3(1-2v_P)}$$

Debye temperature

$$\theta_D = \frac{h}{k_B}\left(\frac{3N}{4\pi V}\right)^{-1/3} v_a$$

Where N is the number of atoms in the unit cell, V is the volume of the unit cell as obtained from Rietveld refinements, and ρ is the density of the sample.

**Calculation of the minimum thermal conductivity**:[2]

Considering the Cahill-Watson-Pohl (CWP) model, where the transport of thermal energy within a material takes place via a random walk to the nearest neighbour of a localized Einstein oscillator, the minimum thermal conductivity at high temperature can be approximated as,

$$\kappa_{min,CWP} \approx 1.21 n^{2/3} k_B \frac{1}{3}(v_L + 2v_T),$$

where *n* is the number density of atoms. Based on the Allen-Feldman theory, the minimum thermal conductivity (diffusive thermal conductivity) at high temperature can be approximated as,

$$\kappa_{diff} \approx 0.76 n^{2/3} k_B \frac{1}{3}(v_L + 2v_T)$$

## Rietveld refinements using powder X-ray diffraction data

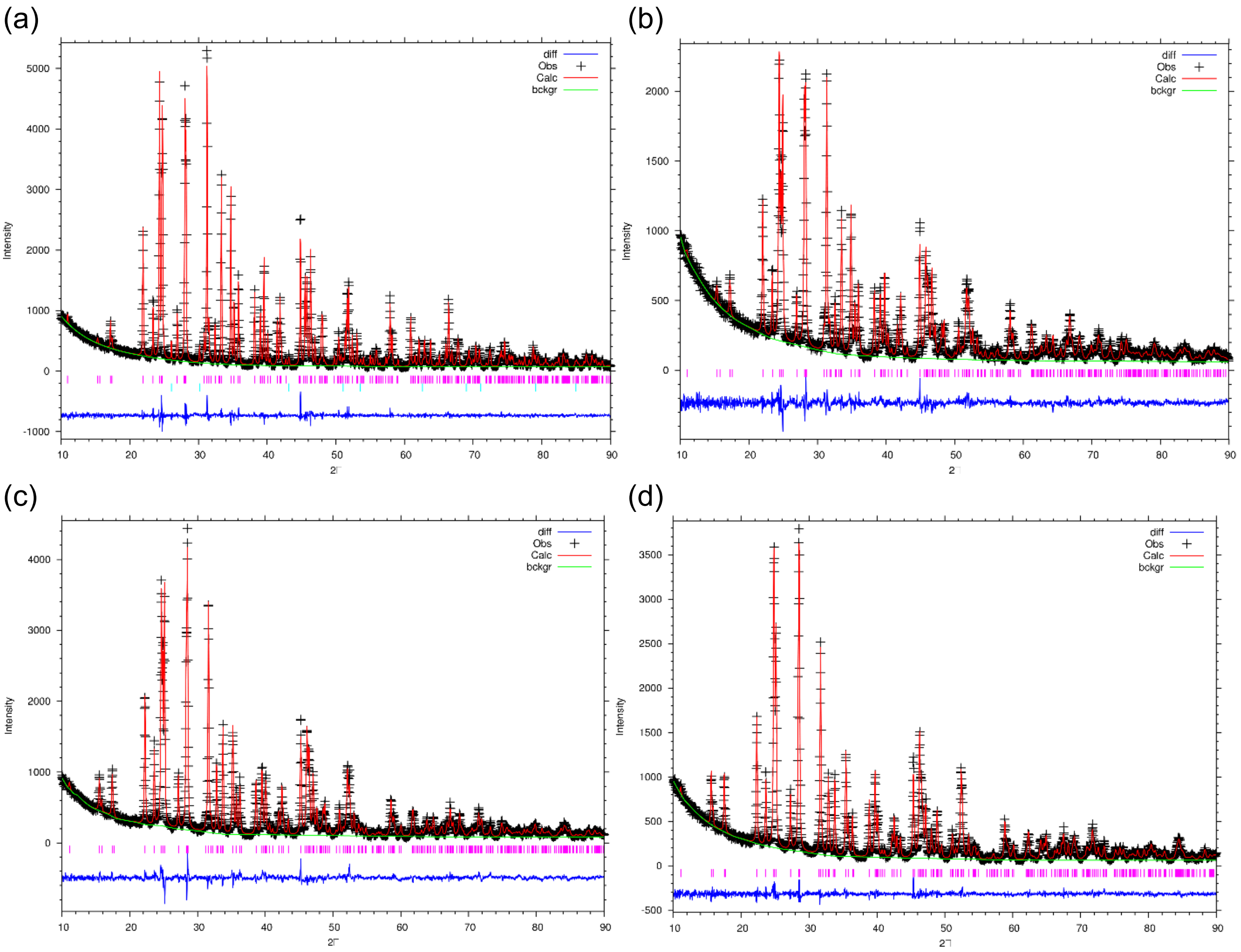


**Figure S1**. Rietveld refinements using powder X-ray diffraction data collected on (a) $x = 0$ ($R_{wp} = 6.6\%$, $\chi^2 = 1.47$), (b) $x = 0.33$ ($R_{wp} = 7.6\%$, $\chi^2 = 1.52$), (c) $x = 0.6$ ($R_{wp} = 7.9\%$, $\chi^2 = 2.17$) and (d) $x = 0.83$ ($R_{wp} = 6.6\ \%$, $\chi^2 = 1.31$). Pink and blue markers correspond to the $Cu_{1-x}Pb_{1-x}Bi_{1+x}S_3$ phase and to PbS, respectively.

**Table S1**. Lattice parameters determined using powder X-ray diffraction data

| Composition | *a*/Å | *b*/Å | *c*/Å |
|---|---|---|---|
| $CuPbBiS_3$ ($x = 0$) | 11.6149(2) | 4.04355(6) | 11.3673(2) |
| $Cu_{0.67}Pb_{0.67}Bi_{1.33}S_3$ ($x = 0.33$) | 11.5648(5) | 4.0364(2) | 11.2701(5) |
| $Cu_{0.4}Pb_{0.4}Bi_{1.6}S_3$ ($x = 0.6$) | 11.4724(4) | 4.0124(1) | 11.2056(4) |
| $Cu_{0.17}Pb_{0.17}Bi_{1.83}S_3$ ($x = 0.83$) | 11.3599(3) | 3.9959(1) | 11.1783(4) |

**Table S2.** Calculated probabilities for the M(4) site in the krupkaite model.

| | |
|---|---|
| P(0) | $(1\text{-}SOF_1)(1\text{-}SOF_2)^2$ |
| P(1) | $SOF_1\ (1\text{-}SOF_2)^2 + 2(1\text{-}SOF_1)SOF_2(1\text{-}SOF_2)$ |
| P(2) | $2SOF_1\ SOF_2(1\text{-}SOF_2) + (1\text{-}SOF_1)SOF_2^2$ |
| P(3) | $SOF_1\ SOF_2^2$ |

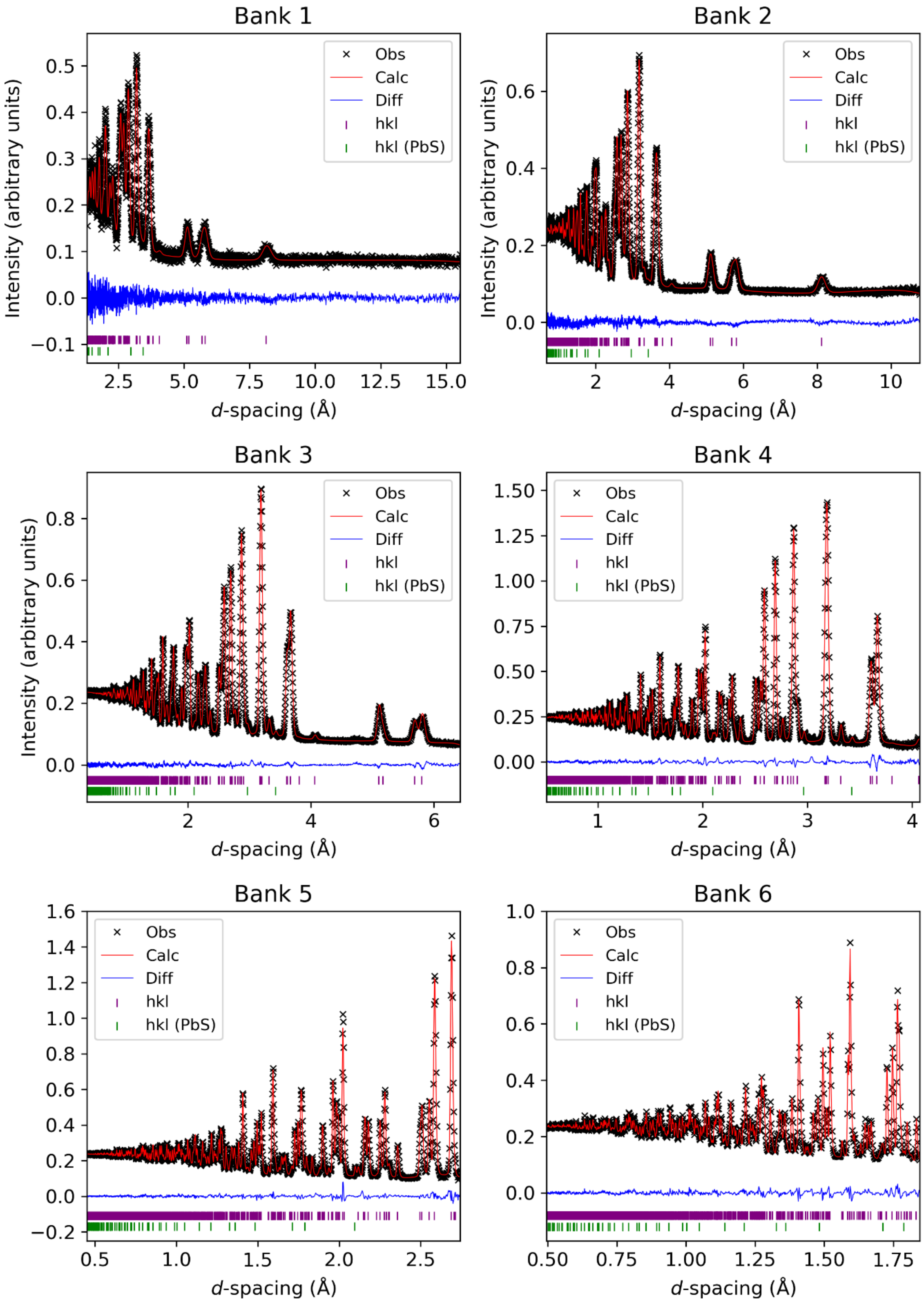


**Figure S2**. Rietveld refinements using neutron diffraction data collected on $CuPbBiS_3$ ($x$ = 0).

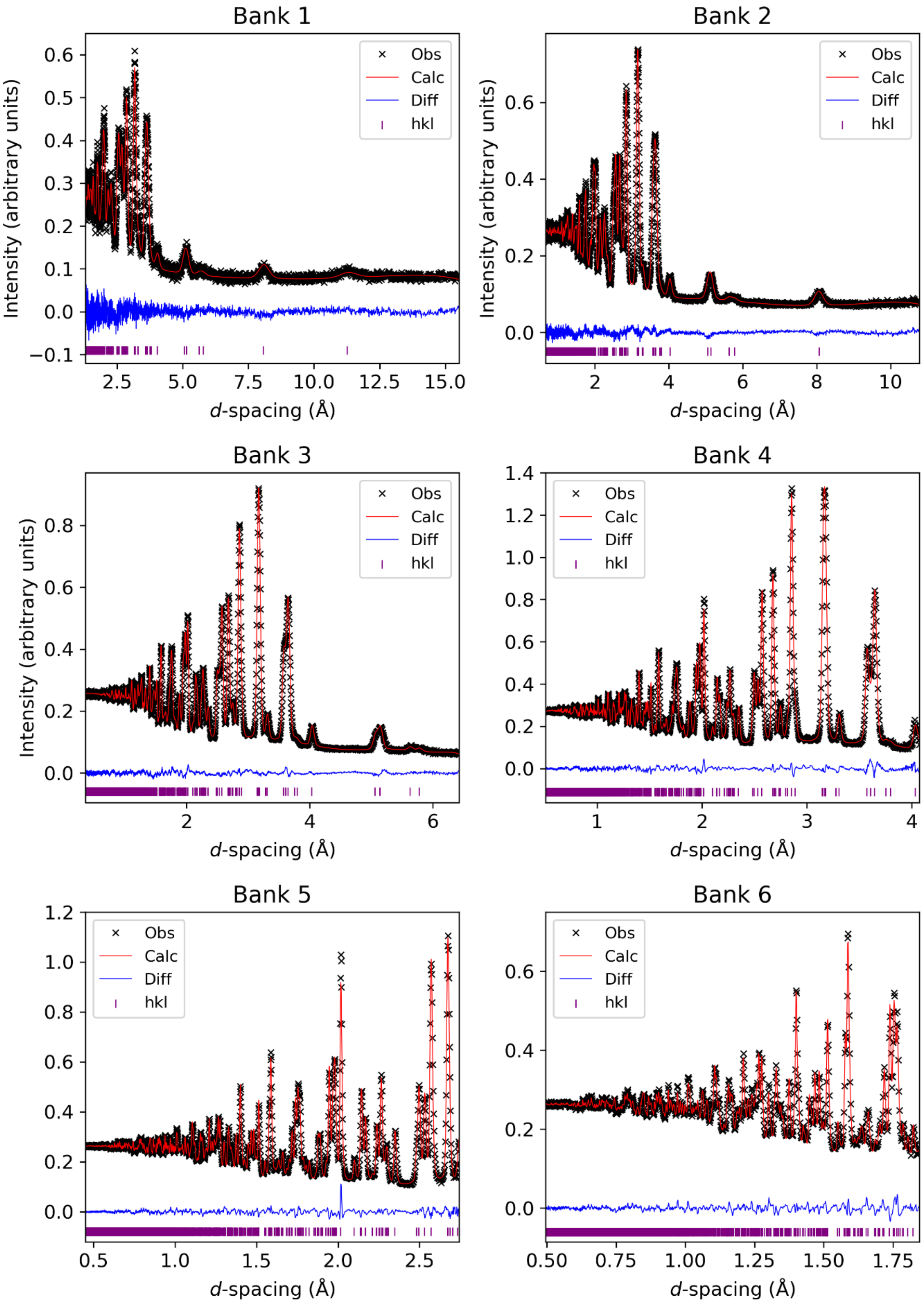


**Figure S3**. Rietveld refinements using neutron diffraction data collected on $Cu_{0.67}Pb_{0.67}Bi_{1.33}S_3$ ($x$ = 0.33)

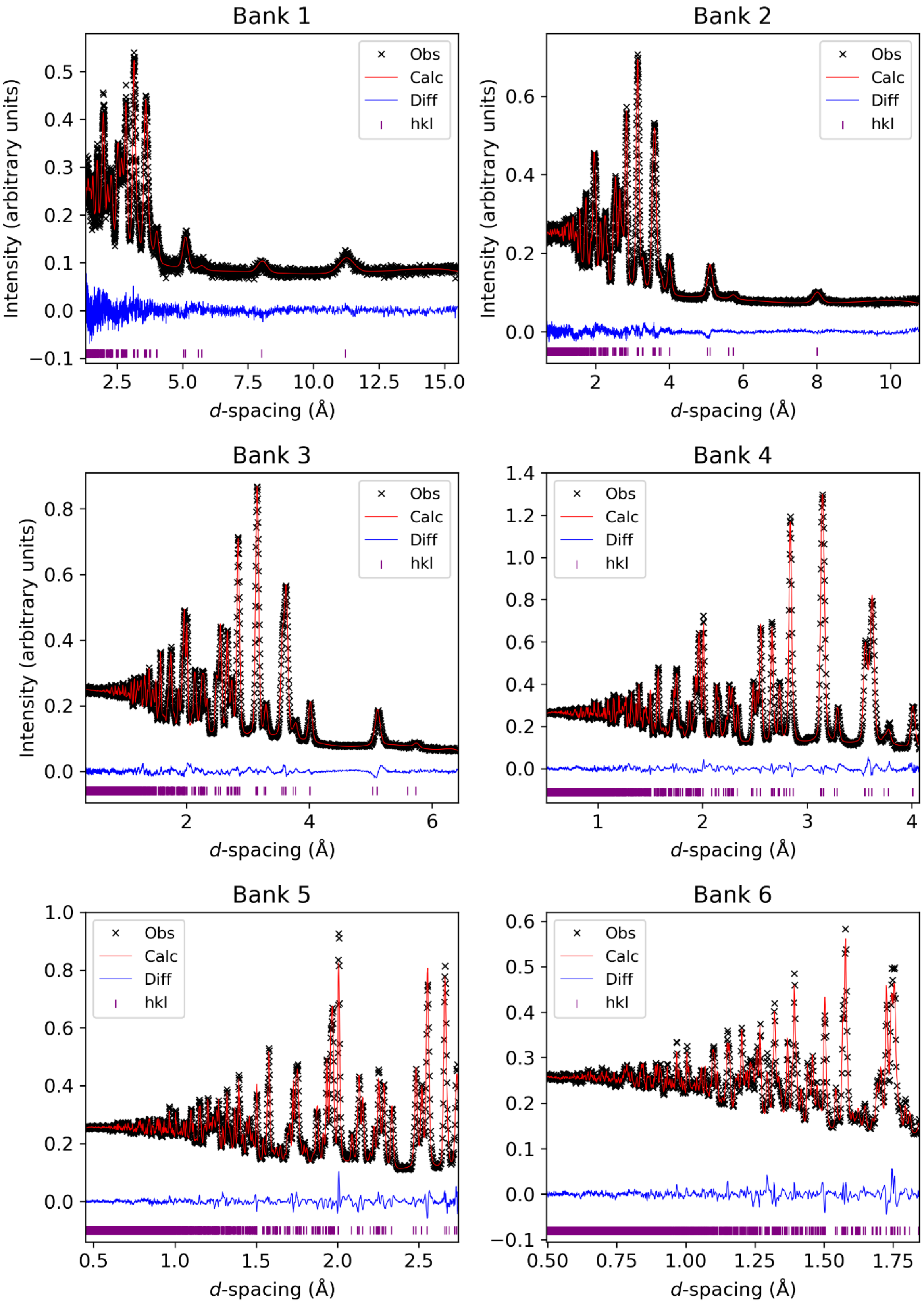


**Figure S4**. Rietveld refinements using neutron diffraction data collected on $Cu_{0.4}Pb_{0.4}Bi_{1.6}S_3$ ($x$ = 0.6).

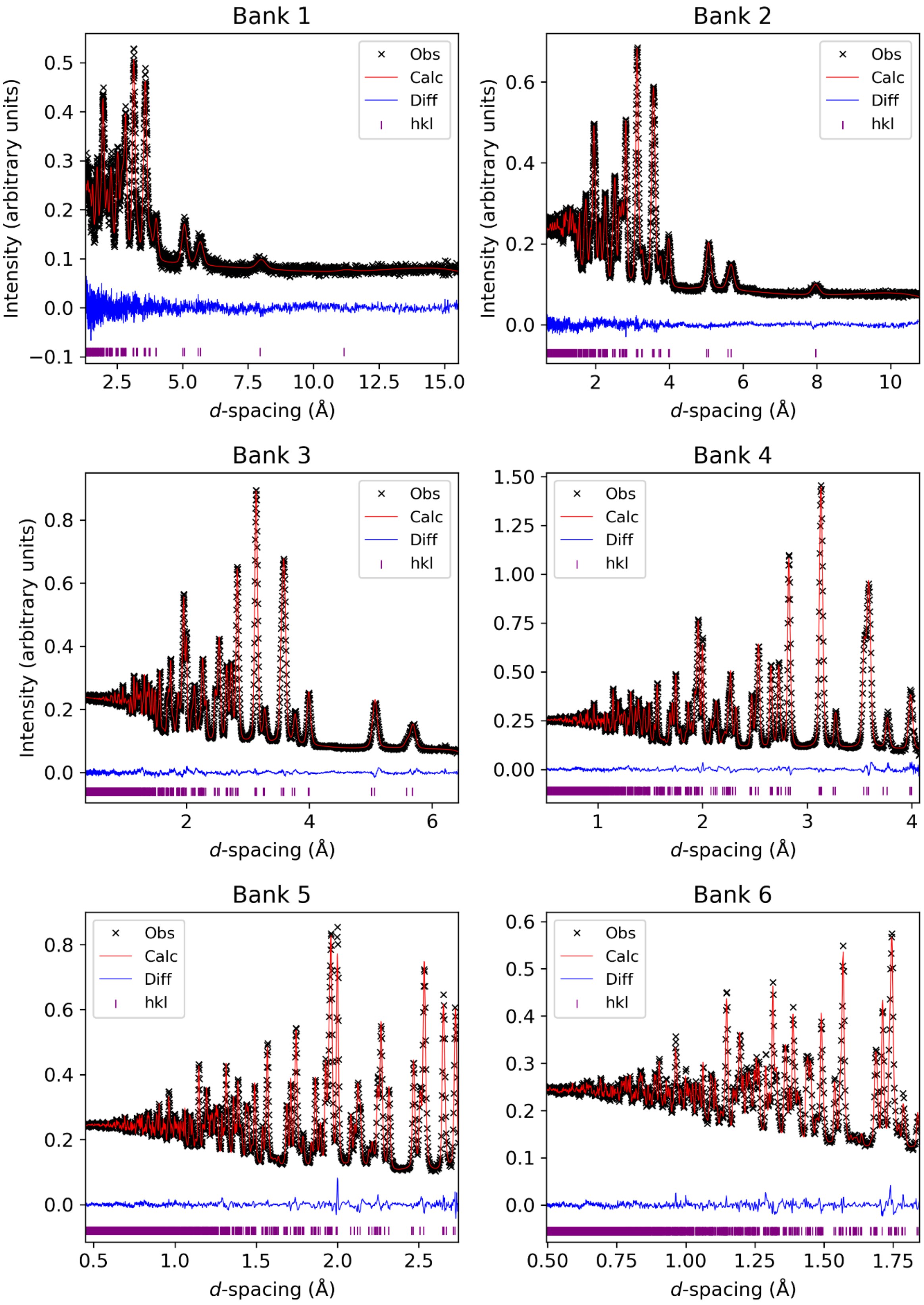


**Figure S5**. Rietveld refinements using neutron diffraction data collected on $Cu_{0.17}Pb_{0.17}Bi_{1.83}S_3$ ($x$ =0.83).

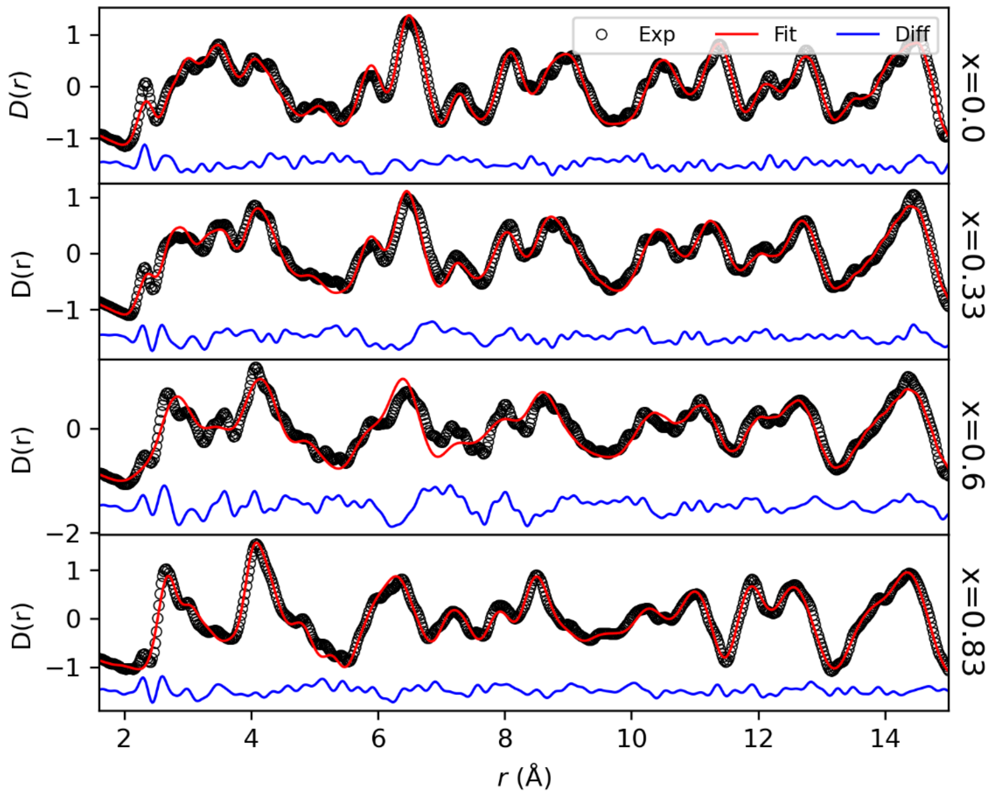


**Figure S6** Small-box model fits of $Cu_{1-x}\square_xPb_{1-x}Bi_{1+x}S_3$ to the aikinite structure ($R_{wp}$ = 18.25, 22.46, 29.24 and 16.89% for *x* = 0, 0.33, 0.6 and 0.83 respectively).

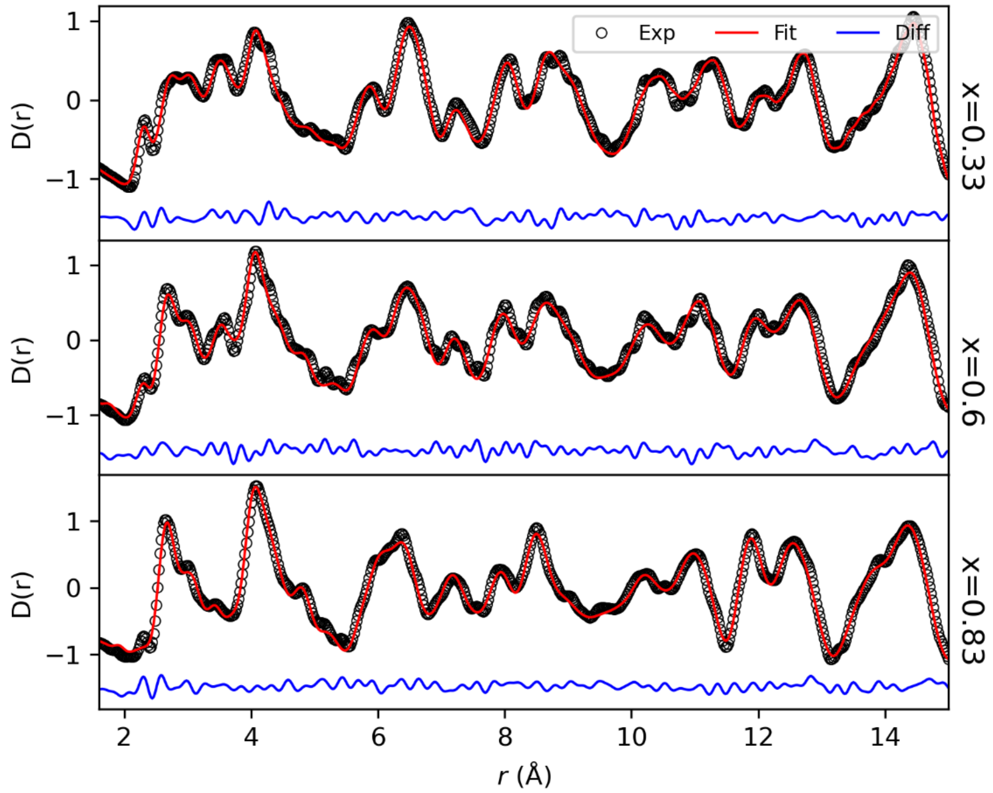


**Figure S7** Small-box model fits of $Cu_{1-x}\square_xPb_{1-x}Bi_{1+x}S_3$ to the krupkaite structure ($R_{wp}$ = 12.61, 14.29 and 11.35% for *x* = 0.33, 0.6 and 0.83 respectively).

**Table S3**. Atomic displacement parameters determined using a small-box krupkaite model.

| x | 0.33 | 0.6 | 0.83 |
| --- | --- | --- | --- |
| **Pb** B / Å$^2$ | 0.98(9) | 1.91(37) | 1.94(43) |
| **Cu** B / Å$^2$ | 0.14(6) | 0.11(9) | 0.00(12) |
| **Bi** B / Å$^2$ | 0.56(6) | 0.47(6) | 0.53(4) |
| **S** B / Å$^2$ | 0.00(5) | 0.00(7) | 0.00(4) |

**Table S4.** Distance window constraints (in Å) for different atom-atom pairs, used for RMC big-box modelling.

| | S-Cu | S-Pb | S-Bi | Cu-Pb | Cu-Bi |
| --- | --- | --- | --- | --- | --- |
| MIN | 2.1 | 2.1 | 2.1 | 2.5 | 2.5 |
| MAX | 2.7 | 3.8 | 3.8 | 4.1 | 4.1 |

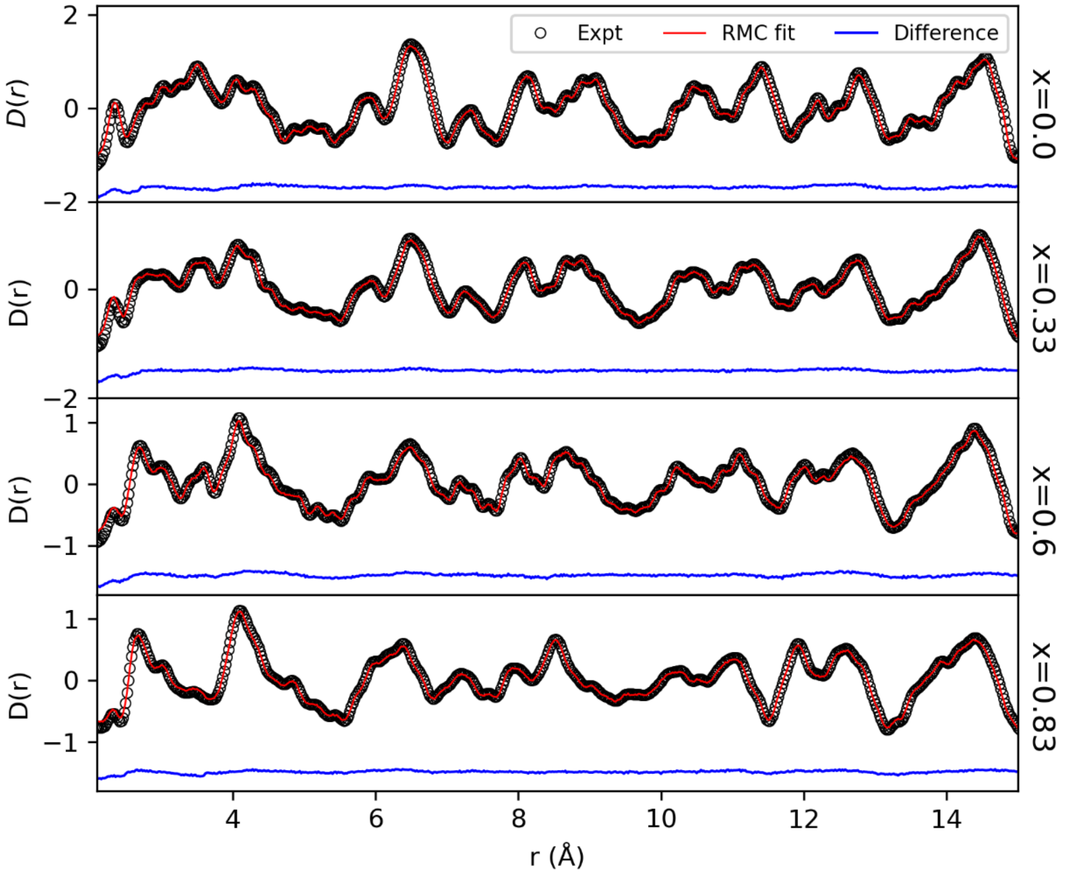


**Figure S8** Big-box model fits of $Cu_{1-x}\square_xPb_{1-x}Bi_{1+x}S_3$. The end member, *x* = 0, is fitted using an aikinite model and the remaining members of the series are modelled using a krupkaite model and atom swapping allowed between symmetry-allowed Cu/□ and Pb/Bi sites.

**Table S5.** $\chi^2$ values for big-box models using a krupkaite model with atom swapping not allowed and allowed.

| x | No atom swapping | Atom swapping allowed |
|---|---|---|
| 0.33 | 17.18 | 14.59 |
| 0.6 | 16.88 | 15.69 |
| 0.83 | 20.11 | 18.31 |

**Table S6**. $Pb^{2+}$ nearest neighbour percentages for sites containing $Cu^+$ and vacancies, calculated from refined krupkaite big-box models.

| x | 3 Pb (%) | 2 Pb (%) | 1 Pb (%) | 0 Pb (%) |
|---|---|---|---|---|
| 0.33 (random) | 22.6 | 55.9 | 21.6 | 0.0 |
| 0.33 (refined) | 22.8 | 55.3 | 21.9 | 0.0 |
| 0.6 (random) | 0.0 | 31.8 | 56.4 | 11.8 |
| 0.6 (refined) | 0.0 | 32.9 | 54.1 | 13.0 |
| 0.83 (random) | 0.0 | 5.7 | 39.7 | 54.7 |
| 0.83 (refined) | 0.0 | 5.7 | 39.5 | 54.7 |

**Table S7**. $Pb^{2+}$ nearest neighbour percentages for **$Cu^+$ cations only**, calculated from refined krupkaite big-box models.

| x | 3 Pb (%) | 2 Pb (%) | 1 Pb (%) | 0 Pb (%) |
|---|---|---|---|---|
| 0.33 (random) | 20.5 | 53.8 | 25.7 | 0.0 |
| 0.33 (refined) | 23.3 | 52.4 | 24.3 | 0.0 |
| 0.6 (random) | 0.0 | 14.7 | 69.2 | 16.1 |
| 0.6 (refined) | 0.0 | 19.6 | 68.8 | 11.6 |
| 0.83 (random) | 0.0 | 5.4 | 38.3 | 56.3 |
| 0.83 (refined) | 0.0 | 7.8 | 41.8 | 50.4 |

**Table S8**. $Pb^{2+}$ nearest neighbour percentages for **vacancies (□) only**, calculated from refined krupkaite big-box models.

| x | 3 Pb (%) | 2 Pb (%) | 1 Pb (%) | 0 Pb (%) |
|---|---|---|---|---|
| 0.33 (random) | 26.7 | 60.0 | 13.3 | 0.0 |
| 0.33 (refined) | 21.7 | 61.4 | 16.9 | 0.0 |
| 0.6 (random) | 0.0 | 43.2 | 47.9 | 8.9 |
| 0.6 (refined) | 0.0 | 41.8 | 44.3 | 13.9 |
| 0.83 (random) | 0.0 | 5.7 | 40.0 | 54.3 |
| 0.83 (refined) | 0.0 | 5.3 | 39.0 | 55.6 |

**Table S9**. $Cu^{+}$ nearest neighbour percentages for $Pb^{2+}$ cations only, calculated from refined krupkaite big-box models.

| x | 3 Cu (%) | 2 Cu (%) | 1 Cu (%) | 0 Cu (%) |
|---|---|---|---|---|
| 0.33 (random) | 26.1 | 46.1 | 24.3 | 3.5 |
| 0.33 (refined) | 39.7 | 29.8 | 20.2 | 10.3 |
| 0.6 (random) | 2.6 | 19.5 | 51.7 | 26.2 |
| 0.6 (refined) | 9.0 | 19.5 | 42.1 | 29.4 |
| 0.83 (random) | 0.2 | 6.7 | 35.1 | 58.0 |
| 0.83 (refined) | 5.8 | 10.3 | 19.6 | 64.3 |

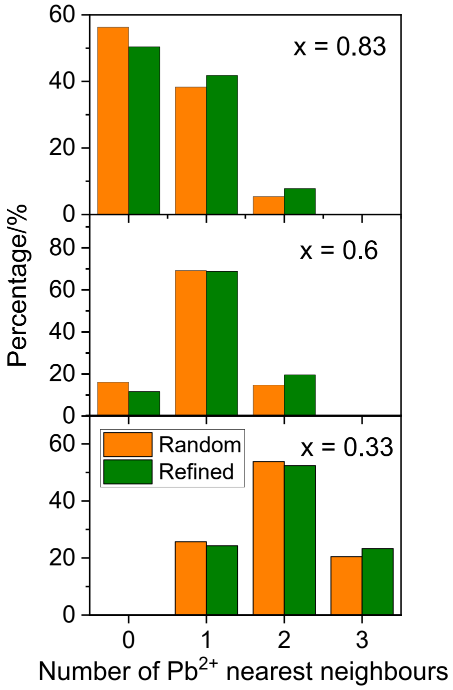


**Figure S9**. Number of $Pb^{2+}$ nearest neighbors around $Cu^{+}$ cations, determined from the initial and refined big-box models of $Cu_{1-x}\square_{x}Pb_{1-x}Bi_{1+x}S_3$, using a krupkaite structural model.

**Table S10.** Estimated average areas of copper-poor and copper-rich regions for each composition.

| Composition | Copper-poor area/Å$^2$ | Copper-rich area/Å$^2$ |
|---|---|---|
| 0.33 | 26 × 15 | 38 × 23 |
| 0.6 | 35 × 20 | 26 × 12 |
| 0.83 | 55 × 30 | 26 × 8 |

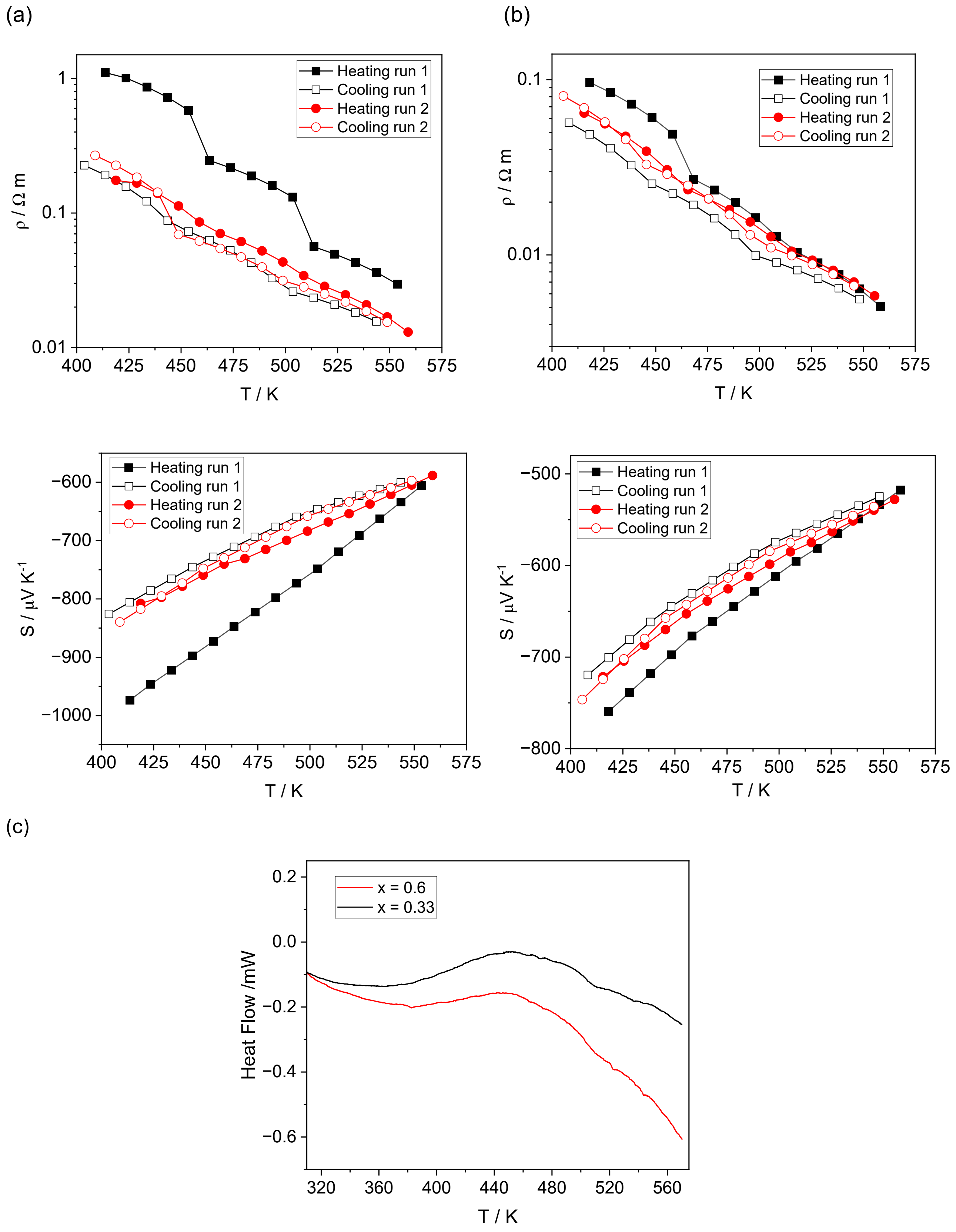


**Figure S10**. (a) Repeated measurements on heating and cooling of the electrical resistivity (top) and the Seebeck coefficient (bottom) for *x* = 0.6. (b) Repeated measurements on heating and cooling of the electrical resistivity (top) and the Seebeck coefficient (bottom) for *x* = 0.83. (c) DSC data collected on heating for x =0.33 and x = 0.6.

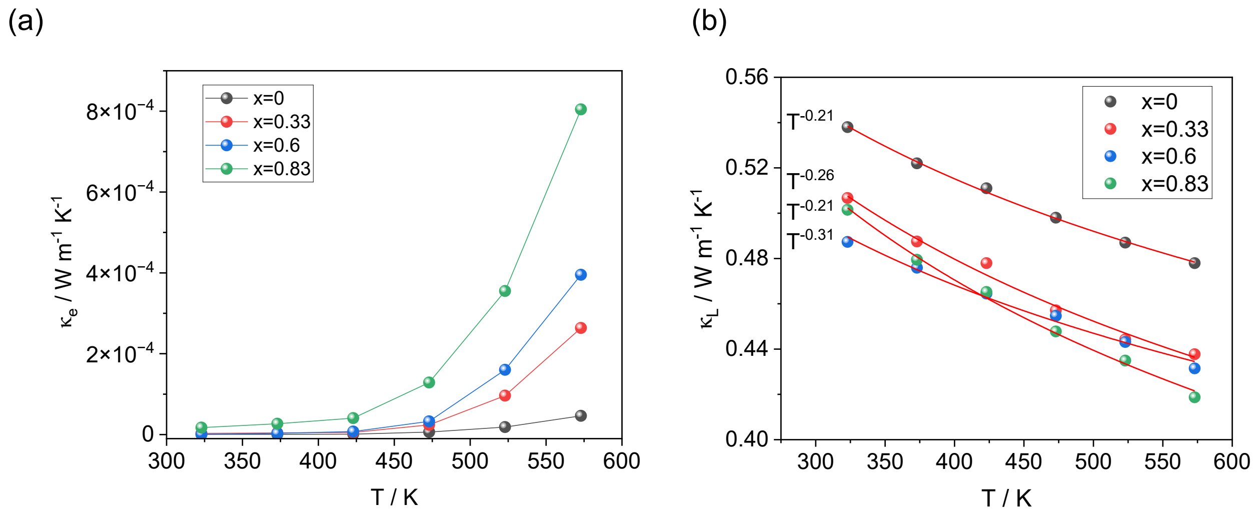


**Figure S11**. (a) Estimated electronic thermal conductivity for $Cu_{1-x}Pb_{1-x}Bi_{1+x}S_3$. (b) Temperature dependence of the lattice thermal conductivity. Red lines illustrate the deviation from the $\kappa_L \propto T^{-1}$ dependence.

**Table S11**. Fitting parameters determined from the lattice thermal conductivity of $Cu_{1-x}Pb_{1-x}Bi_{1+x}S_3$., when using the expression $\kappa_L T = A + \kappa_0 T$.

| *x* | $A$ / W m$^{-1}$ | $\kappa_0$ / W m$^{-1}$ K$^{-1}$ |
|---|---|---|
| 0 | 46(2) | 0.400(3) |
| 0.33 | 54(4) | 0.344(8) |
| 0.6 | 43(4) | 0.360(8) |
| 0.83 | 62(4) | 0.315(9) |

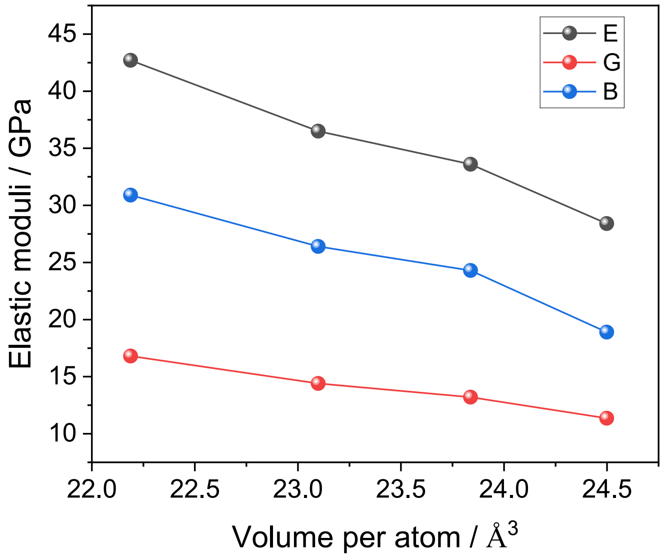


**Figure S12**. Variation of the elastic moduli in the $Cu_{1-x}\square_x Pb_{1-x}Bi_{1+x}S_3$ series with the volume per atom.

**References**

(1) Snyder, G. J.; Snyder, A. H.; Wood, M.; Gurunathan, R.; Snyder, B. H.; Niu, C. Weighted Mobility. *Adv. Mater.* **2020**, *32* (25), 2001537. https://doi.org/10.1002/adma.202001537.

(2) Agne, M. T.; Hanus, R.; Snyder, G. J. Minimum Thermal Conductivity in the Context of Diffuson-Mediated Thermal Transport. *Energy Environ. Sci.* **2018**, *11* (3), 609–616. https://doi.org/10.1039/C7EE03256K.
